# Carbon dioxide utilization in concrete curing or mixing might not produce a net climate benefit

**DOI:** 10.1038/s41467-021-21148-w

**Published:** 2021-02-08

**Authors:** Dwarakanath Ravikumar, Duo Zhang, Gregory Keoleian, Shelie Miller, Volker Sick, Victor Li

**Affiliations:** 1 Center for Sustainable Systems (CSS), School for Environment and Sustainability (SEAS), University of Michigan, Ann Arbor, MI USA; 2 National Renewable Energy Laboratory (NREL), Golden, CO USA; 3 Department of Civil and Environmental Engineering, University of Michigan, Ann Arbor, MI USA; 4 Department of Mechanical Engineering, University of Michigan, Ann Arbor, MI USA

**Keywords:** Environmental impact, Carbon capture and storage

## Abstract

Carbon capture and utilization for concrete production (CCU concrete) is estimated to sequester 0.1 to 1.4 gigatons of carbon dioxide (CO_2_) by 2050. However, existing estimates do not account for the CO_2_ impact from the capture, transport and utilization of CO_2_, change in compressive strength in CCU concrete and uncertainty and variability in CCU concrete production processes. By accounting for these factors, we determine the net CO_2_ benefit when CCU concrete produced from CO_2_ curing and mixing substitutes for conventional concrete. The results demonstrate a higher likelihood of the net CO_2_ benefit of CCU concrete being negative i.e. there is a net increase in CO_2_ in 56 to 68 of 99 published experimental datasets depending on the CO_2_ source. Ensuring an increase in compressive strength from CO_2_ curing and mixing and decreasing the electricity used in CO_2_ curing are promising strategies to increase the net CO_2_ benefit from CCU concrete.

## Abstract (web summary)

Carbon curing or mixing in concrete is promising for carbon dioxide sequestration. Here, the authors show that the increased use of binder material to compensate the loss in compressive strength and electricity for carbon dioxide curing is more likely to increase carbon dioxide emissions on a life cycle basis for carbon cured or mixed concrete.

## Introduction

The capture and utilization of carbon dioxide (CO_2_) to produce economically viable products offers the twin benefit of mitigating climate change and generating economically viable products^1^. Among the portfolio of products that can potentially utilize CO_2_, concrete offers several advantages including (i) a thermodynamically favorable reaction mechanism^2,3^ to sequester the CO_2_ as calcium or magnesium carbonates^4,5^ in ordinary Portland cement (OPC)^6,7^ (ii) the long term CO_2_ sequestration in the form of stable carbonate beyond the life-time of the infrastructure (>60 years) (iii) the significant sequestration potential due to the overall and expected growth in global cement production to meet increasing demand^8^. Concrete along with aggregates and chemicals/fuels are end-products with the potential to sequester the maximum quantity of CO_2_ (in gigatons)^1,3^.

Multiple emerging approaches such as carbonation of recycled concrete aggregates^9^, CO_2_ sequestration in alternative MgO based binders^10^, CO_2_ mineralization in industrial waste-derived aggregates and fillers^11,12^, and CO_2_ dissolution in mixing water^13,14^ have been investigated for CO_2_ utilization in concrete. However, this study focuses on the two approaches of CO_2_ mixing and CO_2_ curing as they are more extensively analyzed and applied for CO_2_ utilization in concrete (Supplementary information (SI) Section 2). In CO_2_ mixing, high-purity CO_2_ is injected into fresh concrete during batching and mixing. The CO_2_ binds to the calcium silicate clinker in OPC to form nano-scale CaCO_3_ particles^15,16^. In CO_2_ curing, CO_2_ is utilized as a curing agent^5^ to accelerate precast concrete fabrication. A review of CO_2_ curing and mixing studies reveals that the CO_2_ uptake potential in CO_2_ curing of precast concrete applications is significantly higher than in CO_2_ mixing (Supplementary Fig. 6).

A common assumption motivating research and commercial interests in CCU concrete is that the CO_2_ uptake during curing and mixing^17–20^ of CCU concrete lowers the CO_2_ burden of concrete production. Estimates show that 0.1–1.4 gigatons of CO_2_ can be utilized in concrete by 2050^1,3^. However, a literature review (SI Section 13) demonstrates these estimates are not based on a comprehensive assessment that accounts for the change in compressive strength of concrete from CO_2_ utilization; the CO_2_ impact of capturing, transporting and utilizing CO_2_; the CO_2_ emissions from compensating for the energy penalty of CO_2_ capture and producing supplementary cementitious materials (SCM), which are by-products of coal electricity and pig iron production; the uncertainty and variability in inventory data and process parameters; and may not always be based on primary experimental data, which is required for a robust life cycle CO_2_ assessment.

CO_2_ curing can decrease the compressive strength of CCU concrete when compared to conventional concrete. For example, a review of 99 experimental datasets from existing literature shows that CCU concrete has a lower compressive strength than conventional concrete in 31 datasets (SI Section 2 Supplementary Fig. 3). In such cases, CCU concrete would require a greater amount of OPC than conventional concrete to produce the same compressive strength. OPC production is a major source of CO_2_ emissions. Therefore, increased OPC content in a concrete formulation leads to an increase in CO_2_ emissions from upstream cement production processes, which may outweigh the benefit of the CO_2_ captured and used in concrete production.

In addition, the CO_2_ impact of CCU concrete can be difficult to generalize due to the lack of consistency in the boundaries and scope of analysis. For example, the energy associated with the capture and transport of CO_2_ is included in certain studies^21^ while being excluded from others^20,22,23^. Moreover, the uncertainty and variability in data and process parameters, which is typical in the early stages of R&D, impacts the environmental assessment of emerging technologies such as CCU concrete^24–28^. Life cycle assessments (LCA) of CCU concrete rely on point values for process parameters rather than parameter distributions that provide a more realistic representation of uncertainty and variability^16,21^. The failure to account for uncertainty in the early stages of technology development can hinder research efforts to address hotspots and increase the CO_2_ benefit from CCU concrete^25,26^. An uncertainty assessment in the early stages of technology development can determine process parameters and inventory items that are the most significant contributors to the CO_2_ burden of CCU concrete and, thereby, help identify research strategies that are most effective in addressing the hotspots.

To address these issues, we review 99 datasets from 19 publications to determine the range of potential net CO_2_ benefit associated with CCU concrete. The net CO_2_ benefit is defined as the difference between the lifecycle CO_2_ impact of producing conventional concrete and producing CCU concrete though CO_2_ curing or CO_2_ mixing. The net CO_2_ benefit accounts for the life cycle CO_2_ impact of the 13 upstream processes to capture, transport and utilize CO_2_ and produce and transport the materials used in concrete. The net CO_2_ benefit accounts for any changes in compressive strength when CCU concrete is produced though CO_2_ curing or CO_2_ mixing. We conduct a sensitivity analysis consisting of a scatter plot analysis and moment independent sensitivity analysis^25,29,30^ to determine the key processes with the most significant influence on the net CO_2_ benefit.

## Results and discussion

### Illustrative example: net CO_2_ benefit for dataset 1

**Fig. 1:**
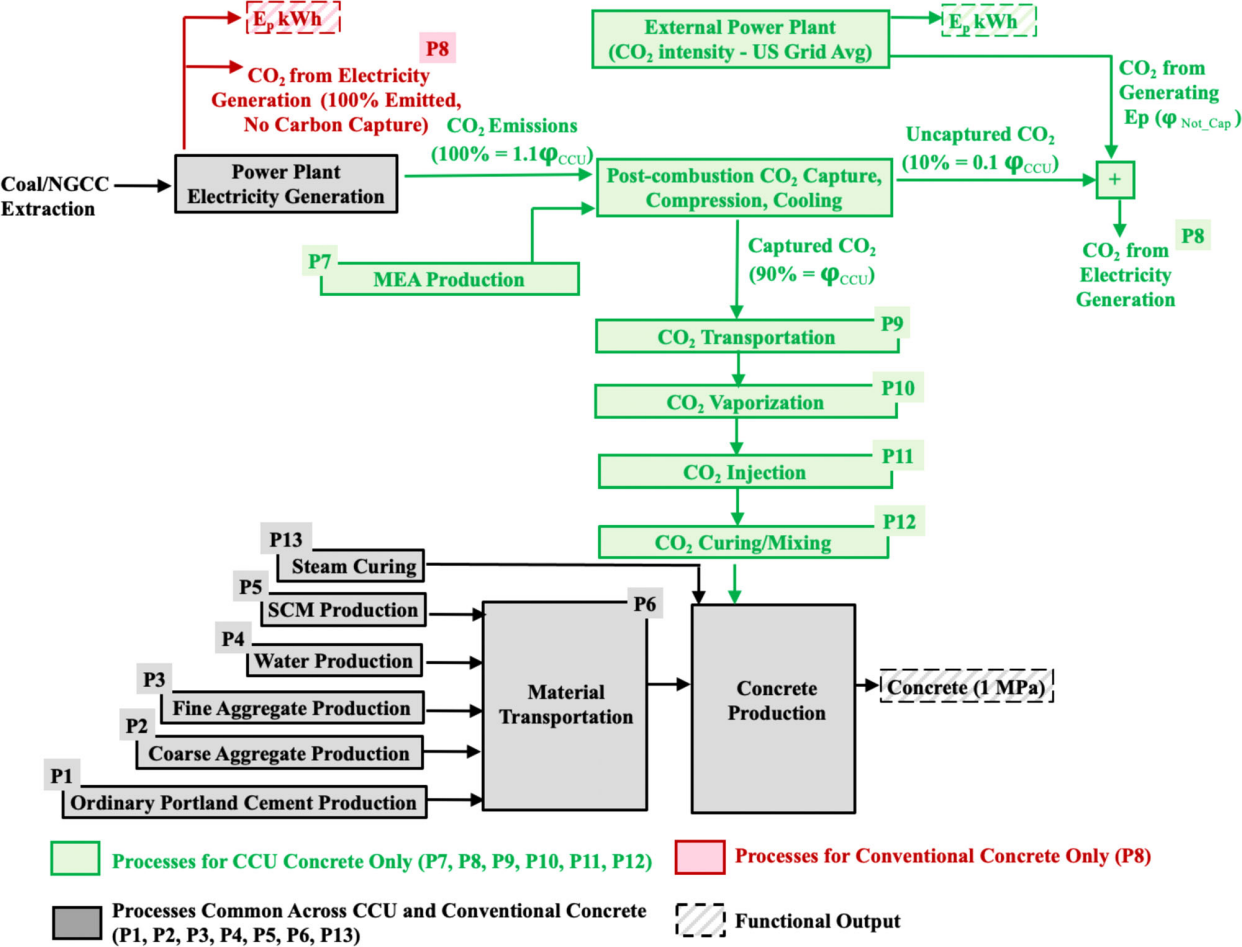
The system boundary diagram depicting the 13 processes, which were accounted for when determining the net CO_2_ benefit of CCU concrete. The processes required to produce CCU concrete are highlighted in gray and green. The processes required to manufacture conventional concrete are highlighted in gray and red. The CO_2_ emissions, which is utilized in the curing or mixing of CCU concrete (φ_CCU_ in kg), is captured from a power plant. The energy penalty from capturing φ_CCU_ (E_p_ kWh) is compensated by an external power plant. When conventional concrete is produced, there is no carbon capture during electricity generation and the CO_2_ from generating E_p_ in the power plant is completely emitted. The functional unit—1 m^3^ of concrete with 1 MPa strength and E_p_ kWh of electricity—is common across the CCU and conventional concrete production pathways. The CO_2_ emissions from each of the CCU and conventional concrete production process is quantified in Eqs. 1 and 5.

We illustrate the interpretation of the results using a single dataset, which helps better understand the findings across the 99 datasets. Consider the dataset of conventional and CCU concrete production in the A1 scenario reported in ref. ^31^ wherein CO_2_ is used for curing and OPC is used as binder. Based on the inventory requirements presented in the dataset, the CO_2_ emissions for the 13 processes (Fig. 1 and Supplementary Table 1), the total life cycle CO_2_ emissions from producing CCU concrete (TOT_CCU_, Eq. 1) and conventional concrete (TOT_Conv_, Eq. 5), and the net CO_2_ benefit (Eq. 6) are stochastically determined in 10,000 Monte Carlo runs.

**Fig. 2:**
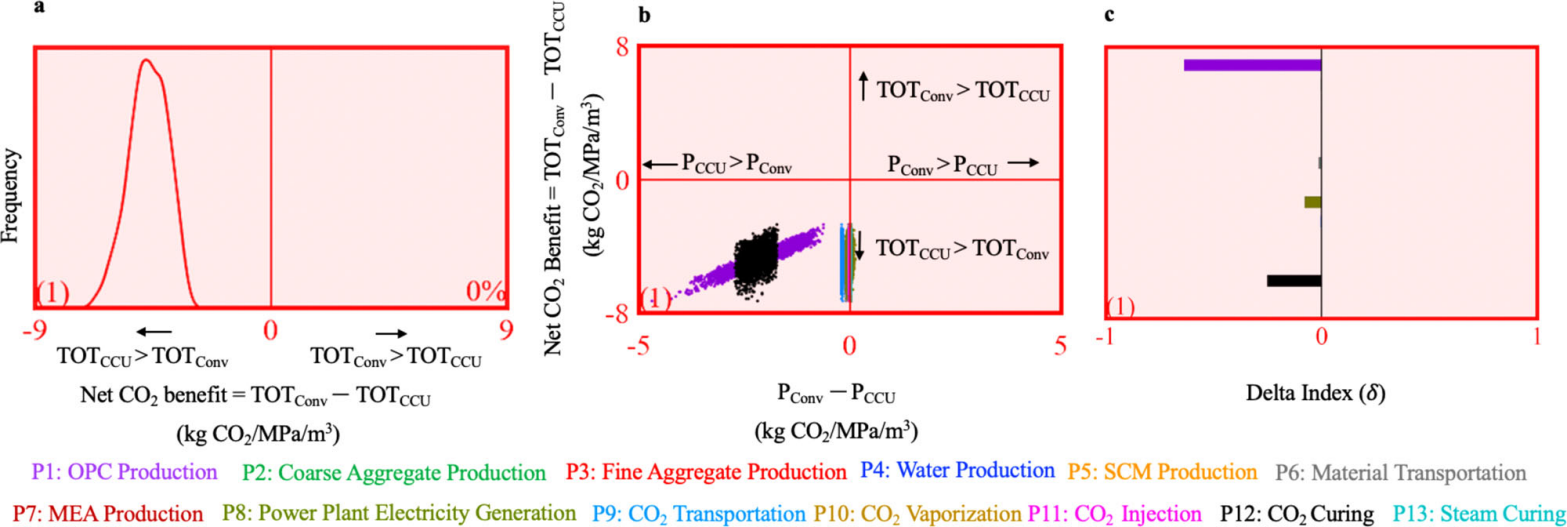
Illustrative example to explain the interpretation of the results. **a** The distribution of the net CO_2_ benefit of CCU concrete in 10,000 Monte Carlo runs. The net CO_2_ benefit is the difference between the total CO_2_ emissions from producing conventional concrete (TOT_Conv_) and CCU concrete (TOT_CCU_) **b** the scatter plot demonstrating the sensitivity of the net CO_2_ benefit to the difference in the CO_2_ emissions from the 13 contributing processes for conventional concrete (P_Conv_) and CCU concrete (P_CCU_). Positive values on the y-axis indicates that TOT_Conv_ > TOT_CCU_ (upper right and upper left quadrants), negative values on the *y*-axis indicate that TOT_CCU_ > TOT_Conv_ (lower right and lower left quadrants), positive values on the *x*-axis indicate that P_Conv_ > P_CCU_ (upper right and lower right quadrants), negative values on the *x*-axis indicate that P_CCU_ > P_Conv_ (upper left and lower left quadrants) **c** delta indices to determine the sensitivity of the net CO_2_ benefit to the difference between the CO_2_ emissions from P_Conv_ and P_CCU_. The number in parenthesis represents the dataset number (from the literature review) for which the results are determined. The red background in (**a**–**c**) signifies that the net CO_2_ benefit is negative (i.e., TOT_CCU_ is greater than TOT_Conv_) in at least 5000 out of the 10,000 Monte Carlo runs (i.e., likelihood greater than 50%). If the background is green, then it signifies that the net CO_2_ benefit is positive with a likelihood greater than 50%.

The distribution in Fig. 2a quantifies the likelihood of the net CO_2_ benefit in dataset 1 being positive (right of the *y*-axis) in 10,000 Monte Carlo runs. If the net CO_2_ benefit is positive, then the total life cycle CO_2_ emissions from producing CCU concrete (TOT_CCU_) is lower than conventional concrete (TOT_Conv_). For dataset 1 the likelihood is 0%, which signifies that TOT_CCU_ is greater than TOT_Conv_ in all of the 10,000 Monte Carlo runs. Alternately, the distribution for the net CO_2_ benefit is always negative which indicates that, on a life cycle basis, producing CCU concrete is more CO_2_ intensive than conventional concrete in the 10,000 Monte Carlo runs.

The scatter plot analysis of the 10,000 Monte Carlo runs (Fig. 2b) determines the key drivers in the relationship between the difference in the CO_2_ emissions from the 13 conventional concrete (P_Conv_) and CCU concrete (P_CCU_) processes and the net CO_2_ benefit. The scatter plot shows the 10,000 net CO_2_ benefit values on the ordinate and the 10,000 values of the difference between the CO_2_ emissions from the 13 processes on the abscissa. A visual inspection of the scatter plot reveals a difference in the slopes of the scattered points. A higher slope indicates higher sensitivity of the net CO_2_ benefit to the contributing process. The scatter plot for dataset 1 (Fig. 2b) demonstrates that the difference in the CO_2_ emissions from cement used (P1:OPC) has a significant slope. As the difference in CO_2_ emissions from OPC production are scattered in the lower left quadrant, the CO_2_ emissions from OPC production for CCU concrete is greater than conventional concrete. Therefore, for dataset 1, the difference between the CO_2_ emissions from OPC production is the most important reason for TOT_CCU_ being greater than TOT_Conv_ (i.e., the net CO_2_ benefit being negative).

This finding is confirmed by a moment independent sensitivity analysis^25,29,30^, which determines δ indices for each of the 13 processes contributing to the net CO_2_ benefit (Fig. 2c). The δ index is a measure of the contribution from the difference in the CO_2_ emissions from a conventional and CCU concrete production process to the probability distribution function of the net CO_2_ benefit. The process with a greater δ index value has a greater contribution to the net CO_2_ benefit than a parameter with a lower δ index value. By convention, if the δ value is negative then the CO_2_ emissions from the process are greater in CCU concrete production than in conventional concrete production. The difference in the CO_2_ emissions from OPC production has the highest δ value and is, therefore, the most significant contributor to the net CO_2_ benefit. The negative value indicates that the CO_2_ emissions from OPC production are greater in CCU concrete than in conventional concrete. The finding from the sensitivity analysis for dataset 1 can be attributed to the compressive strength of CCU concrete (16–17.4 MPa) being lower than conventional concrete (18–18.6 MPa). The mean compressive strength of CCU concrete (16.7 MPa) is 9% lower than that of conventional concrete (18.3 MPa). This implies that in dataset 1 a greater mass of OPC is produced for CCU concrete to achieve the same compressive strength as conventional concrete. In dataset 1, the OPC produced per MPa for CCU concrete is 24.8 kg/MPa and for conventional concrete is 22.6 kg/MPa.

Using a mean value of 0.948 kg CO_2_/kg OPC for the life cycle CO_2_ footprint of OPC (Supplementary Table 2), the CO_2_ emissions from OPC production for CCU concrete is 23.5 kg CO_2_/MPa and for conventional concrete is 21.4 kg CO_2_/MPa. Therefore, the difference between the CO_2_ emissions from OPC production for CCU and conventional concrete is 2.1 kg CO_2_/MPa, which is greater than the CO_2_ utilized in CCU concrete (1.1 kg CO_2_/MPa, SI Section 1). As a result, the life cycle CO_2_ emissions from the increased OPC production in CCU concrete is greater than the CO_2_ utilized for curing the CCU concrete.

### Increased binder use undermines CO_2_ benefit of CCU concrete

**Fig. 3:**
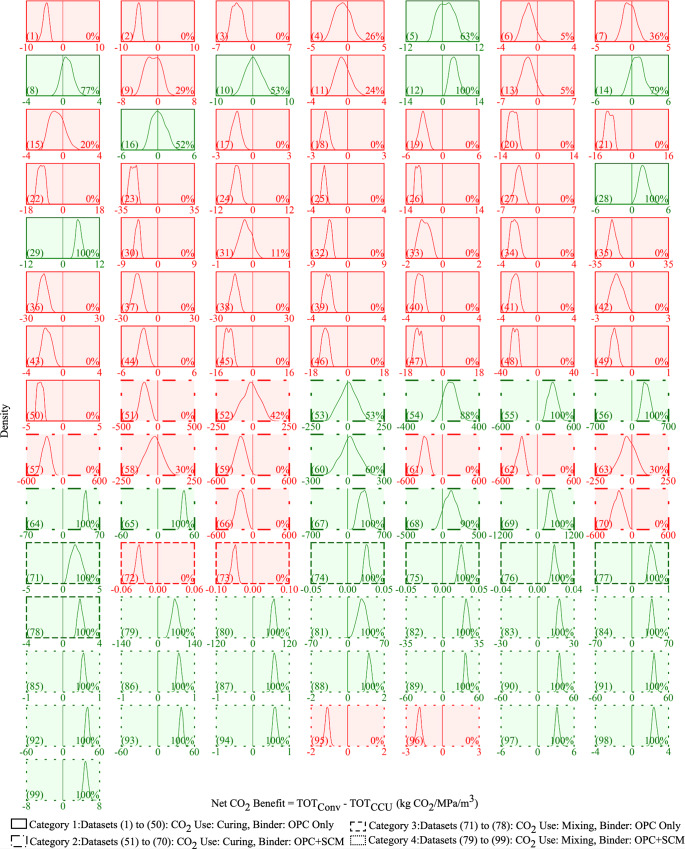
The net CO_2_ benefit of CCU concrete production across 99 datasets. The curve in each plot represents the distribution of the net CO_2_ benefit, which is the difference between the total CO_2_ emissions from producing conventional and CCU, across 10,000 Monte Carlo runs. When the net CO_2_ benefit is negative in at least 5000 of the 10,000 Monte Carlo runs (50% likelihood), the background is red. If the background is green, then it signifies that the net CO_2_ benefit is positive with a likelihood greater than 50%. The likelihood is presented as a percentage value in the lower right corner of the plot. The parenthesized value in the lower left corner is the dataset number.

We extend the analysis conducted for dataset 1 to the remaining 98 datasets (Fig. 3). The 99 datasets are divided into 4 categories. Category 1 has 50 datasets wherein CO_2_ is used for curing and the binder consists of only OPC. Category 2 has 20 datasets wherein CO_2_ is used for curing and the binder consists of a mix of OPC and supplementary cementitious materials (SCM). Category 3 has 8 datasets wherein CO_2_ is used for mixing and the binder consists of only OPC. Category 4 has 21 datasets wherein CO_2_ is used for mixing and the binder consists of a mix of OPC and SCM.

**Fig. 4:**
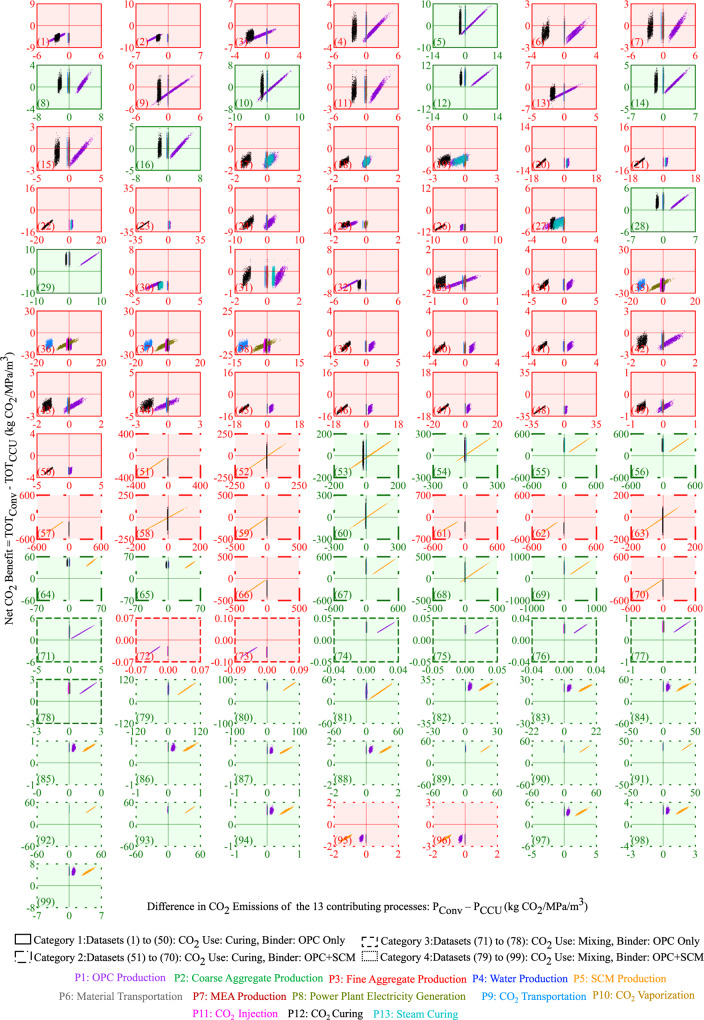
Scatter plot analysis to determine the impact of the 13 processes on the net CO_2_ benefit across the 99 datasets. The difference between the CO_2_ emissions from the 13 processes of CCU and conventional concrete production is plotted on the *x*-axis and the net CO_2_ benefit is plotted on the *y*-axis. The parenthesized value in the lower left corner is the dataset number. Higher resolution scatter plots for each of the 13 processes can be downloaded from SI Section 9 Supplementary Table 12.

**Fig. 5:**
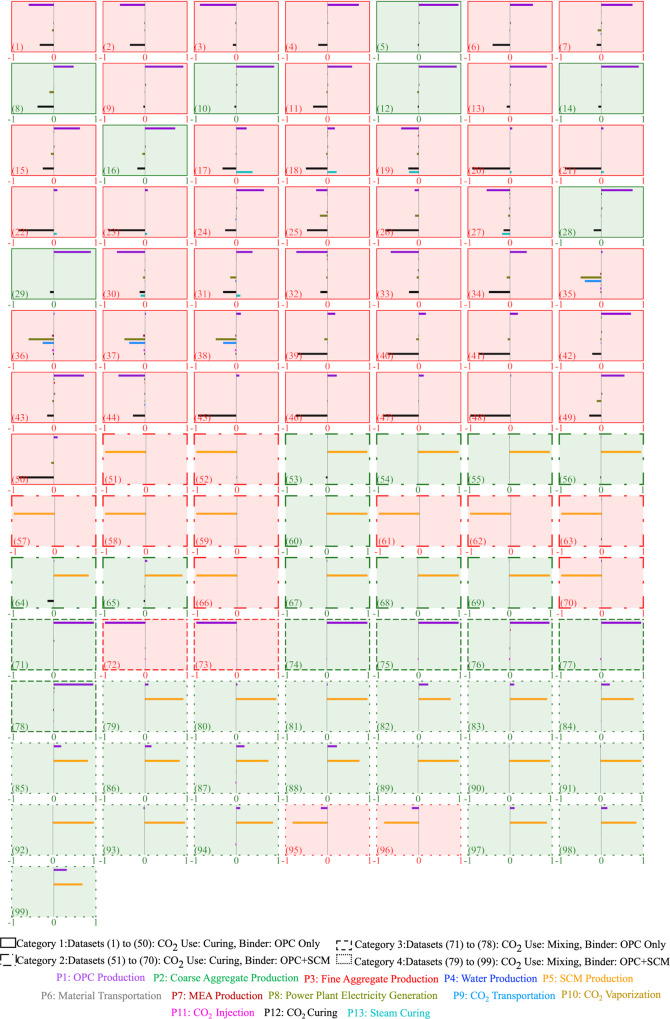
δ indices quantifying influence of the difference between the CO_2_ emissions from the 13 processes of CCU and conventional concrete production on the net CO_2_ benefit. A process with a greater δ index value has a greater influence on the net CO_2_ benefit than a process with a lower δ index value. The parenthesized value in the lower left corner is the dataset number.

A visual inspection of the slopes in the scatter plot in Fig. 4 and the δ indices in Fig. 5 reveal that the net CO_2_ benefit is most sensitive to the amount of OPC produced and used in the design mix (e.g., P1 in datasets 1, 2, 3, and 4 in Fig. 5), the energy used for CO_2_ curing (e.g., P12 in datasets 21, 22, and 23 in Fig. 5) and SCM produced and used (e.g., P5 in datasets 51, 52, 53, and 54 in Fig. 5).

The plots with the red background in Figs. 3,  4, and  5 demonstrate that the net CO_2_ benefit is negative (i.e., CCU concrete has higher life cycle CO_2_ emissions than conventional concrete) with at least a 50% likelihood in 56 out of the 99 datasets. The compressive strength in CCU concrete decreases due to CO_2_ curing when compared to conventionally cured concrete. The OPC and SCM consumed to produce the same compressive strength is greater in CCU concrete than in conventional concrete. Therefore, the results demonstrate the CO_2_ burden of increased OPC and SCM consumption for CCU concrete outweighs the benefit of the CO_2_ that is captured and used in CCU curing. Additionally, in category 1 datasets, the electricity use in the CO_2_ curing process is the second key contributor to the increase in the total life cycle CO_2_ emissions from CCU concrete (e.g., datasets 46, 47, and 48 in Fig. 5).

The results in Fig. 5 also demonstrate that the life cycle CO_2_ emissions from capturing, compressing, transporting and vaporizing CO_2_, and the CO_2_ emissions from producing fine aggregate, coarse aggregate, water and steam curing are not significant contributors to the net CO_2_ benefit. This can be attributed to the mass of CO_2_ utilized in concrete being lower than the mass of the cement and coarse and fine aggregate (Supplementary Fig. 1) and the life cycle CO_2_ intensity of coarse and fine aggregate being significantly lower than cement (Supplementary Table 2).

To investigate the change in results when CO_2_ intensity of OPC production decreases, we determine the net CO_2_ benefit of CCU concrete when CO_2_ is captured from a cement plant (SI Section 11). The results show that CCU concrete has higher life cycle CO_2_ emissions than conventional concrete (i.e., negative CO_2_ benefit) in 44 out of the 99 datasets (Supplementary Fig. S4 in SI Section 11) when compared to 56 out of the 99 datasets in the baseline scenario (Fig. 5). Therefore, when CO_2_ is captured from a cement plant, there is a lower likelihood of CCU concrete producing a negative net CO_2_ benefit than when CO_2_ is captured from a power plant. The difference in the results can be attributed to the reduced CO_2_ intensity of cement production due to CO_2_ capture at the cement plant.

### Results across CO_2_ use, binder type, and allocation method

**Fig. 6:**
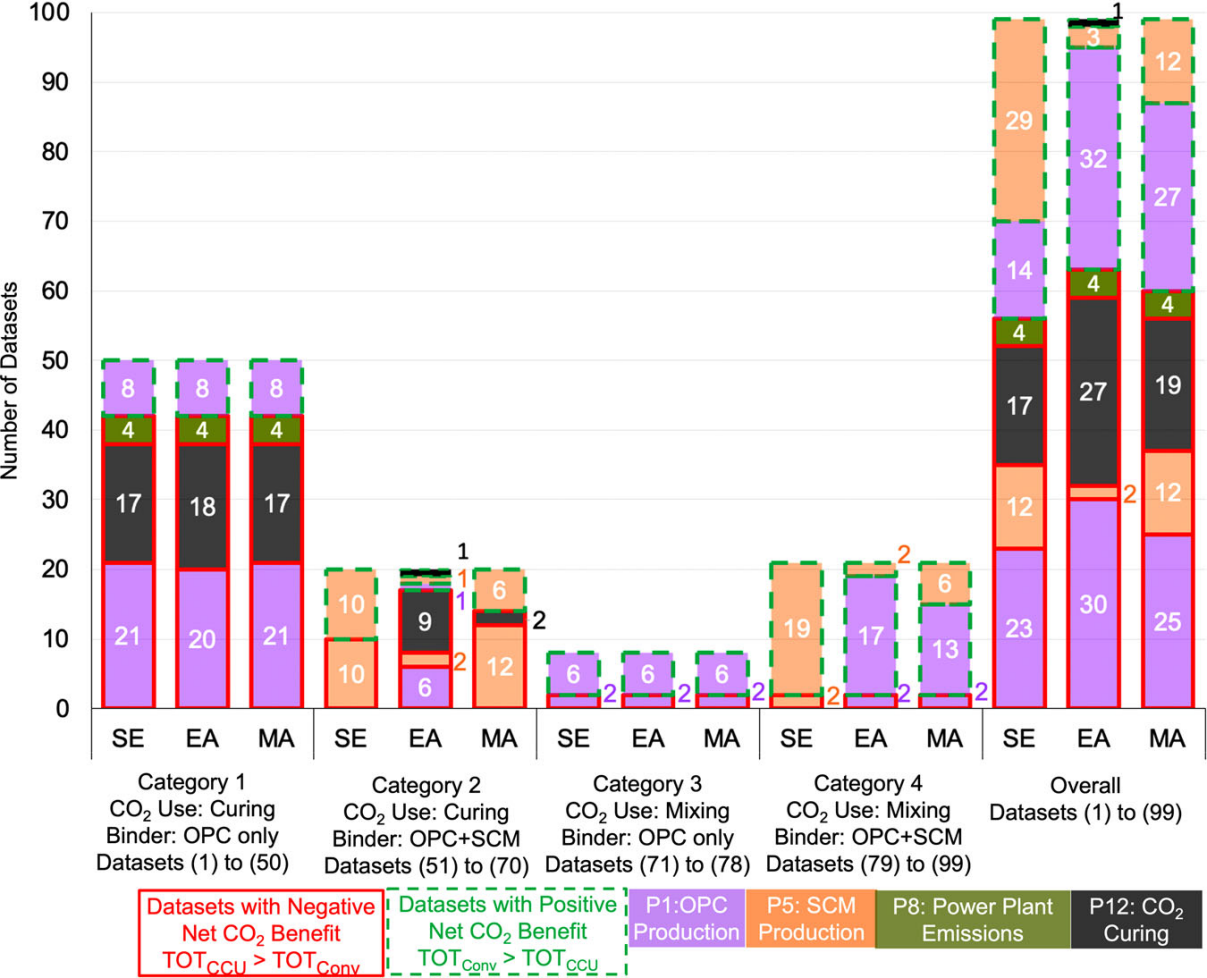
Results providing a break-up of the datasets with positive and negative net CO_2_ benefit from CCU concrete and the most significant driver of the net CO_2_ benefit of CCU concrete across category 1, category 2, category 3, and category 4 datasets (SI Section 2). The results were determined using system expansion (SE), economic value-based (EA), and mass-based (MA) allocation to determine the CO_2_ emissions from slag (co-product of iron ore production) and fly ash (co-product of coal electricity generation), which are used as SCM in category 2 and 4 datasets.

Fig. 6 summarizes the results depending on whether CO_2_ is used for curing or mixing, SCM is used as a binder material or not, and the type of allocation method used to determine the CO_2_ emissions from producing the SCM material. Two types of SCMs are used in concrete production—ground granulated blast furnace slag and fly ash. Slag is a by-product of pig-iron production and fly ash is a by-product of electricity generation in coal power plants. As a result, we need a method to allocate total CO_2_ emissions between slag and pig-iron and between fly-ash and coal electricity. We use three methods—system expansion (SE), mass-based (MA), and economic value-based allocation (EA)—to allocate and account for the life cycle CO_2_ emissions from producing the SCMs (process P5, SI Sections 4, 5, and 6).

The overall results in Fig. 6 demonstrate that in 36 (EA in “Overall”) to 43 (SE in “Overall”) of the 99 datasets reported in the literature, CCU concrete production has a lower life cycle CO_2_ emission than conventional concrete production. In these cases, CCU concrete substituting conventional concrete lowers CO_2_ emissions. Negative CO_2_ net benefit values are obtained in the remaining 56 to 63 datasets. A similar analysis for CO_2_ capture from a NGCC power plant shows that the net CO_2_ benefit is negative in 61, 65, and 68 of the 99 datasets when SE, MA, and EA are used, respectively (SI Section 10). The overall results demonstrate that the CO_2_ benefit of CCU concrete production is negative in 56–68 of the 99 datasets depending on whether CO_2_ is captured from a coal or a NGCC power plant and when SE, MA or EA is used. As a result, there is a higher likelihood of the net CO_2_ benefit of CCU concrete being negative. Consequently, not all CCU concretes can help realize sequestering 0.1 to 1.4 gigatons/year of CO_2_ in concrete by 2050^1,3^.

The results of the sensitivity analysis are similar across categories 1, 2, 3, and 4 as the production and use of binder material (OPC in categories 1 and 3 and OPC + SCM in Categories 2 and 4) has the most significant influence on the net CO_2_ benefit.

The overall results on the number of datasets in which there is a positive net CO_2_ benefit (i.e., CCU is less CO_2_ intensive than conventional concrete) is not significantly impacted by the choice of system expansion SE, MA or EA. CCU is less CO_2_ intensive than conventional concrete in a minimum of 36 datasets when EA is used and a maximum of 43 datasets when SE is used. However, for category 2 and 4 datasets, the choice of SE, MA or EA impacts the results from the δ indices sensitivity analysis. When SE or MA is used in category 2 datasets, SCM use is a key contributor to the net CO_2_ benefit of CCU concrete. When EA is used in category 2 datasets, the CO_2_ emissions from the CO_2_ curing process is the key contributor. When SE is used in category 4 datasets, SCM use is a key contributor to the difference in the total CO_2_ emissions between conventional and CCU concrete. When EA or MA is used in category 4 datasets, OPC use is the key contributor. However, the choice of SE, MA or EA is an artifact of the method of analysis—not actually changing real CO_2_ emissions—and, therefore, is not within the purview of engineering strategies to improve processes and decrease the CO_2_ emissions from producing CCU concrete.

The findings from this analysis are based on the design mixes, material usage, compressive strength, and parameters such as the CO_2_ curing duration, water to cement and SCM to cement ratios obtained from the 99 datasets (SI Section 2 Supplementary Figs. 1 to 5). It is important to note that the findings do not preclude future research (as discussed below) from optimizing the design mixes, curing processes and material properties to increase the net CO_2_ benefit from CCU concrete.

### Strategies to improve the net CO_2_ benefit of CCU concrete

An R&D agenda focused on the following items, which are within the control of the CCU concrete production process, can increase the net CO_2_ benefit.

(i) Ensure increase in compressive strength from CO_2_ curing: a key priority is to determine a CO_2_ curing protocol that consistently increases the compressive strength of CCU concrete. An increase in compressive strength implies that a smaller quantity of carbon intensive binder material is used in CCU concrete to achieve the same compressive strength as conventional concrete (i.e., lower quantity of OPC or SCM is consumed on a kg per MPa basis). Fine tuning the curing process such as duration of the pre-hydration and post-carbonation water compensation are promising candidates to restore the reduction in 28-day compressive strength observed for CO_2_-cured concrete^32–34^. For example, a longer duration of pre-hydration may enhance 28-day compressive strength but decreases CO_2_ uptake at early age. Further investigations are needed for enhancing consistency of CO_2_-cured concrete production and for implementing the laboratory strategies in field applications.
(ii) Decrease the CO_2_ emissions from the CO_2_ curing process: electricity use, which is the key contributor to the CO_2_ emitted during the CO_2_ curing process, can be lowered by streamlining the curing process. Future research can investigate and standardize promising options, such as natural drying or waste heat drying for pre-curing of CO_2_-cured concrete^35^ with the end goal of accelerating adoption in industry.
(iii) Improve understanding on the impact of CO_2_ curing on durability: the findings of this research are based on the compressive strength property of CCU concrete, which is limited from a lifecycle perspective. Prior studies show that construction and repair frequencies are key drivers in determining concrete life cycle CO_2_ impacts^36,37^. Therefore, the effect of CCU on concrete durability must be considered when analyzing life cycle CO_2_ emissions. Preliminary lab scale studies demonstrate that CO_2_ curing improves durability related parameters such as permeability, sorptivity and sulfate and acid resistance^22,38–40^. However, the variability in the curing conditions and the design mixes used in the studies should be accounted for to ensure that durability gains can be consistently realized when CO_2_ curing of concrete is adopted at a commercial scale. Future work can prioritize standardizing the CO_2_ curing protocol (e.g., the steam curing time, pre-hydration time, post-hydration time), and study the resulting durability impact on different design mixes (e.g., use of different SCMs), with the overall goal of identifying optimal curing conditions and design mixes to maximize durability. This applies to ready-mix concrete and general precast applications with end-products such as masonry units^32,35^, pipes^22^, and pavers^41^. In addition, CO_2_ curing for reinforced concrete needs further investigation due to the possibility of increased risk of steel reinforcement corrosion led by concrete carbonation. Moreover, CCU can be potentially synergized with established strategies for concrete crack width control, e.g., engineered cementitious composites with microfiber reinforcement, to further promoting concrete durability^42,43^.

The system boundary (Fig. 1) assumes that the CO_2_ captured from the power plant is used for CCU concrete production without any intermediate storage. In practice, the total CO_2_ captured from a power plant may be significantly greater than the maximum utilization capacity at a CCU concrete production plant. In such cases, the excess captured CO_2_ may be temporarily stored for future utilization in CCU concrete production or routed towards other utilization pathways. Given that CO_2_ utilization is an emerging field and in the early stages of commercialization, there is a lack of time-sensitive data on how the captured CO_2_ feedstock is either temporarily stored or immediately allocated to other utilization pathways. As a result, a system boundary that incorporates the time-sensitive utilization of CO_2_ captured from a power plant is beyond the scope of this work and is a topic for future research.

The transport of CO_2_ through pipelines has a lower CO_2_ impact than road-based transport using semi-trailer trucks^44^, which is modeled in this analysis. To quantify the maximum possible gains from shifting to a less carbon intensive mode of CO_2_ transport, we conduct a scenario analysis with the optimistic assumption that the CO_2_ impact of CO_2_ transportation is zero (SI Section 12). Despite this optimistic assumption of zero-carbon CO_2_ transport, CCU concrete has a lower CO_2_ impact than conventional concrete in 44 of the 99 datasets, which is similar to the 43 of the 99 datasets obtained in the baseline scenario (Supplementary Fig. 10 versus Fig. 5). As a result, a shift from road to pipeline based CO_2_ transport will not impact the findings from this analysis.

This analysis focusses on the use of pure CO_2_ and two approaches of CO_2_ utilization—curing and mixing—as they are more extensively investigated (e.g., 99 datasets used in this study) than alternate approaches such as concrete curing with flue gas^21,45–47^, carbonation of recycled concrete aggregates^9^, CO_2_ sequestration in alternative MgO based binders^10^, and CO_2_ dissolution in mixing water^13,14^. The increased availability of experimental data is necessary to robustly quantify the net CO_2_ benefit of CCU concrete and account for the impact of data uncertainty and process variability on the results. For example, further experimental research can generate data on the variability in the compressive strength of flue-gas cured concrete properties when the 2-week curing time is reduced^47^ and the CO_2_ concentration in the flue gas is varied^45^. With increased availability of inventory and process data from future experimental research, the life cycle approach presented in this study can be extended to quantify the net CO_2_ benefit of CCU concrete produced from flue gas and other alternate approaches of CO_2_ utilization.

The impact of CO_2_ curing or CO_2_ mixing on the lifetime behaviors and geography specific factors are important practical considerations when using CCU concrete in commercial applications. The lifetime behaviors are impacted by the variations in the geographical sources of raw materials, mix type, product type and the service environment in which the concrete is deployed. For example, the impact of CO_2_ curing on concrete lifespan would differ substantially between concrete with and without steel reinforcement, due to the heightened steel corrosion^48^ caused by the CO_2_-induced pH reduction^49^. Additionally, geography specific factors such as sulfate-rich soils^50^, cold regions^39^, or acidic environments^22^ can impact the durability of CCU concrete. The findings of this study can be further complemented by future research which quantifies the impact of variations in the lifetime and the geography specific factors on the net CO_2_ benefit of CCU concrete.

## Methods

### Literature review to categorize CO_2_ use in concrete

We conducted a literature review to obtain the 99 datasets from 19 studies presenting life cycle material and energy inventory data and process parameters for the production of CCU and conventional concrete. The literature review identified the 19 studies^16,19,22,23,31–33,35,38,40,51–59^ as they were the only ones to report the following three items (i) the design mix consisting of the energy and material inventory required for the production of conventional and CCU concrete (SI Section 2). The energy and material inventory are required to determine the life cycle CO_2_ impact of producing conventional and CCU concrete; (ii) the quantity of CO_2_ used in mixing or curing of concrete. This is required to determine the life cycle CO_2_ impact of capturing, transporting, and utilizing the CO_2_ used in producing CCU concrete; and (iii) the compressive strength of CCU and conventional concrete at the end of 28 days, which helps account for the change in the material property between conventional and CCU concrete. The 28-day compressive strength is among the most widely used technical parameters for assessing concrete quality, categorizing concrete mix designs^60^ and forms the basis for concrete structural design^61,62^ and is, therefore, chosen as the functional property based on which conventional and CCU concrete are compared. Based on whether CO_2_ is used in CCU concrete for curing or mixing and if SCM was used in the design mix, the 99 datasets were organized into four categories.

(i) Category 1: CO_2_ is used in the curing of concrete and only OPC is used as the cementitious material in the design mix^22,31,33,38,40,56–59^. This category contains 50 datasets.
(ii) Category 2: CO_2_ is used in the curing of concrete and a combination of OPC and SCM is used as the cementitious material in the design mix^23,32,35,55^. This category contains 20 datasets.
(iii) Category 3: CO_2_ is used in the mixing of concrete and only OPC is used as the cementitious material in the design mix^16,19,51^. This category contains 8 datasets.
(iv) Category 4: CO_2_ is used in the mixing of concrete and a combination of OPC and SCM is used as the cementitious material in the design mix^16,51–54^. This category contains 21 datasets.

SCM took the form of either ground granulated blast furnace slag, which is a by-product of the pig-iron production^63^, or fly ash, which is a by-product of electricity generation in coal power plants.

### Functional Unit

The use of CO_2_ during mixing or curing changes the compressive strength of CCU concrete when compared to concrete produced through conventional mixing or curing. In addition, an energy penalty (E_p_ kWh) is incurred for CCU concrete in power plants due to the energy associated with capturing the CO_2_, which is used in the curing or mixing of CCU concrete (φ_CCU_, kg CO_2_). E_p_ is not incurred when conventional concrete is produced since there is no CO_2_ capture. Therefore, the net CO_2_ benefit of substituting CCU concrete for conventional concrete should account for the CO_2_ impact from the change in the compressive strength and E_p_, which is incurred in power plants only when CO_2_ is captured.

As a result, we use a functional unit of concrete with 1 MPa compressive strength and 1 m^3^ of volume and E_p_ kWh of electricity.

The functional unit accounts for the change in compressive strength and ensures consistency by normalizing the materials and energy consumed for producing 1 m^3^ of CCU and conventional concrete to 1 MPa of compressive strength. The inclusion of E_p_ kWh of electricity in the functional unit accounts for the difference in CO_2_ emissions from electricity generation without CO_2_ capture in the conventional concrete pathway and with CO_2_ capture in the CCU concrete pathway. E_p_ is determined based on the mass of CO_2_ captured from the power plant (Supplementary Table 1 Process 8).

### CCU concrete production—system boundary and CO_2_ emissions

The literature review revealed that the total life cycle CO_2_ emissions from producing CCU concrete is the sum of the CO_2_ emissions from 13 key processes required to capture, transport, and utilize CO_2_ and produce the materials required in the design mix of concrete (Fig. 1).

The expression used to determine the total life cycle CO_2_ emissions from producing CCU concrete based on the CO_2_ emissions from the 13 processes is presented in Eq. 1. The 13 expressions within parenthesis in Eq. 1 correspond to the CO_2_ emissions from the 13 processes (Fig. 1).

1 $${\mathrm{TOT}}_{{\mathrm{CCU}}} 	= \, \left({{\upvarphi}_{\mathrm{C}} \ast {\mathrm{C}}_{{\mathrm{CCU}}}} \right) + \left({{\upvarphi}_{{\mathrm{CA}}} \ast{\mathrm{CA}}_{{\mathrm{CCU}}}}\right) + \left( {{\upvarphi}_{{\mathrm{FA}}} \ast{\mathrm{FA}}_{{\mathrm{CCU}}}} \right) + \left({{\upvarphi}_{\mathrm{W}} \ast {\mathrm{W}}_{{\mathrm{CCU}}}} \right)\\ \, 	\quad{\,\,}+\left({{\upvarphi}_{{\mathrm{SCM}}} \ast{\mathrm{SCM}}_{{\mathrm{CCU}}}}\right) + \left({{\mathrm{D}}_{\mathrm{M}} \ast {\upvarphi}_{{\mathrm{TM}}} \ast{\mathrm{M}}_{{\mathrm{Conv}}}} \right)\\ \, 	\quad{\,\,}+\left( {{\upvarphi}_{{\mathrm{CCU}}} \ast{\mathrm{j}}_{{\mathrm{MEA}}}} \right) +\left({{\mathrm{Alloc}}_{{\mathrm{elec}}} \ast{\upvarphi}{\mathrm{Not}}\;{\mathrm{Cap}} + {\upvarphi}_{{\mathrm{Avg}}}\ast{\mathrm{E}}_{\mathrm{p}}} \right)\\ \, 	\quad{\,\,}+ \left({{\upvarphi}_{{\mathrm{CCU}}} \ast \left( {1 +2{\mathrm{T}}_{\mathrm{w}}}\right) \ast {\mathrm{D}}_{{\mathrm{CO2}}}\ast {\upvarphi}_{\mathrm{T}}} \right)\\ \, 	\quad{\,\,}+ \left({\upvarphi}_{{\mathrm{CCU}}} \ast{\upvarphi}_{{\mathrm{Vap}}}\right) +\left({\upvarphi}_{{\mathrm{CCU}}} \ast \left({\upvarphi}_{{\mathrm{Inj}}} +\left(1 - \upeta \right)\right)\right)\\ \, 	\quad{\,\,}+\left({\upvarphi}_{{\mathrm{CO2}}\_{\mathrm{Cur}}}\right) +\left({\upvarphi}_{{\mathrm{Stm}}\_{\mathrm{Cur}}}\right)$$

Process 1 to 4—Ordinary Portland cement (C), coarse aggregate (CA), fine aggregate (FA), and water (W) production: The CO_2_ impact is the product of (i) the life cycle CO_2_ emissions from producing the material (φ_C_, φ_FA_, φ_CA_ and φ_W_ in kg CO_2_/kg material) and (ii) and the mass of material used in the design mix normalized to the compressive strength of CCU concrete (C_CCU_, CA_CCU_, FA_CCU_ and W_CCU_ in kg material/MPa/m^3^). The material used and the compressive strength are obtained from the literature review (SI Section 2) and φ_C_, φ_FA_, φ_CA_, and φ_W_ are obtained from the ecoinvent database (Supplementary Table 2).

Process 5—SCM production: SCM_CCU_ represents the mass of SCM used in the design mix normalized to the compressive strength of CCU concrete (in kg material/MPa/m^3^).

Slag and fly ash, which are co-products of iron-ore production and electricity generation from coal, are used as SCM in the design mix of concrete. Three methods—system expansion (SE), economic value-based allocation (EA) and mass-based allocation (MA)—are widely used in LCA to determine the CO_2_ emissions of co-products being generated by a single system.

In SE, the CO_2_ emissions from producing a required mass of slag is determined by expanding the system to include the production of a corresponding mass of iron-ore (based on a ratio of iron-ore to slag, SI Section 4). In the case of MA and EA, the total CO_2_ emission from a process producing both iron-ore and slag is allocated between iron-ore and slag based on the mass and economic value of the co-products, respectively (SI Sections 5 and 6). To explore variability in the CO_2_ emissions from CCU concrete production based on the allocation method, this analysis applies the three methods when determining the CO_2_ emissions for slag and fly ash.

The CO_2_ impact of slag (φSCM_slag in kg CO_2_/kg slag) is determined from Eq. 2

2 $$\upvarphi _{{\mathrm{SCM}}\_{\mathrm{slag}}} = {\mathrm{Alloc}}_{{\mathrm{slag}}} * {\mathrm{7}}{\mathrm{.7}} * \upvarphi _{{\mathrm{IO}}}$$

The value of Alloc_slag_ is 1, 0008. or 0.11 when SE, MA or EA is chosen, respectively (SI Sections 4, 5 and 6).

φ_IO_ is the life cycle CO_2_ emissions from producing 1 kg of iron ore and is 2.2 kg CO_2_/kg iron ore (SI Section 4).

When fly ash is used as the SCM, the CO_2_ impact per kg of fly ash (φ_SCM_ash_ in kg CO_2_/kg fly ash) is determined from Eq. 3

3 $$\upvarphi _{{\mathrm{SCM}}\_{\mathrm{ash}}} = {\mathrm{Alloc}}_{{\mathrm{ash}}} * {\mathrm{22}}{\mathrm{.7}} * \upvarphi _{{\mathrm{Elec}}\_{\mathrm{Coal}}} * \upalpha _{{\mathrm{Cap}}}$$

The value of Alloc_ash_ is 1, 0.02 or 0.06 when SE, MA or EA is chosen, respectively (SI Sections 4, 5 and 6). φ_Elec_Coal_, which is the life cycle CO_2_ emission from producing 1 kWh of coal electricity, is 1.25 kg CO_2_/kWh (SI Section 4). α_Cap_ is 0.1 if CO_2_ is captured at a coal plant and used in CCU concrete production. α_Cap_ is 1 if there is no carbon capture at a coal plant i.e., when CO_2_ is captured from a combined cycle natural gas plant and used in CCU concrete production.

Process 6—Material Transportation: The CO_2_ emissions from material transport is the product of the 5 materials used in the design mix (M_CCU_ in kg/MPa/m^3^), the CO_2_ intensity of the mode of transportation used (φ_M_ in kg CO_2_ per kg-km) and the distance over which the materials are transported (D_M_ in km). M_CCU_ represents C_CCU_, FA_CCU_, CA_CCU_, W_CCU_ and SCM_CCU_ from processes 1 to 5. D_M_ values for road, rail, ocean and barge transport are obtained from the national average values for the US concrete industry (SI Section 7)^60^. φ_M_ for the four transportation modes are obtained from the Ecoinvent database (SI Section 7).

Process 7—Monoethanolamine (MEA) Production: The CO_2_ impact of carbon capture is the product of the mass of CO_2_ which is captured and used in the curing or mixing of CCU concrete (φ_CCU_, kg CO_2_) and the life cycle CO_2_ emissions from producing a monoethanolamine (MEA) post-combustion CO_2_ capture system (φ_MEA_). φ_MEA_ is obtained from the literature review of 21 studies^44,64–83^ (SI Section 3).

MEA systems are considered as they capture CO_2_ with a high efficiency (90%)^64,65,84^, capture CO_2_ from dilute concentrations^85^, are retrofittable to power plants currently in operation and are a commercially mature technology^86,87^. The power sector accounts for 28% of the overall CO_2_ emissions in the U.S^88^ and is, therefore, a good candidate for carbon capture. As a result, we consider CO_2_ capture from power plants. Post-combustion capture is considered as it more commonly deployed than oxy-fuel and pre-combustion systems^65,85^. The reader can refer to^65,85^ for further details on the underlying physical principles of carbon capture using MEA, which is beyond the scope of this work.

Process 8—Power plant electricity generation: When CCU concrete is produced, the total CO_2_ emissions from the power plant is the sum of two components.

$$\left( {{\mathrm{Alloc}}_{{\mathrm{elec}}} * \upvarphi _{{\mathrm{Not}}\;{\mathrm{Cap}}} + \upvarphi _{{\mathrm{Avg}}} * {\mathrm{E}}_{\mathrm{p}}} \right)$$

Alloc_elec_ quantifies the allocation of CO_2_ emissions from a coal power plant between the co-products of electricity and fly ash, which is used as SCM in concrete production in certain datasets. Alloc_elec_ is 0.98 or 0.94 as economic or mass allocation allocates 0.02 and 0.06 of the total CO_2_ emissions from the coal power plant to the co-product of fly ash (SI Sections 5 and 6). Alloc_elec_ is 1 when electricity is sourced from a combined cycle natural gas power plant or when system boundary expansion is used (instead of economic or mass allocation). φ_Not Cap_ accounts for the 10% of CO_2_ which is not captured as the capture efficiency of the MEA system is 90%^64,65,84^.

The second component accounts for the CO_2_ emissions from compensating for the energy penalty (E_p_ in kWh), which is incurred when CO_2_ is captured from a power plant. The second component is the product of E_p_ and the CO_2_ intensity of the electricity used to compensate for E_p_ (φ_Avg_ in kg CO_2_/kWh).

E_p_ is quantified as follows

4 $${\mathrm{E}}_{\mathrm{p}} = \upvarphi _{{\mathrm{CCU}}} * \left[ {\left( {{\mathrm{heat}}_{{\mathrm{ccu}}} * {\mathrm{hte}} * {\mathrm{0}}{\mathrm{.277}}} \right) + {\mathrm{E}}_{{\mathrm{pump}}} + {\mathrm{E}}_{{\mathrm{liq}}}} \right]$$

φ_CCU_ is the mass of CO_2_, which is captured from a power plant and utilized in CCU concrete production. heat_ccu_ represents the heat required to regenerate the MEA (2.7 to 3.3 MJ/kg CO_2_, Supplementary Table 5), which could have alternately been used to generate electricity in the power plant^70,89–91^. hte is the heat to electricity factor (0.09 to 0.25, Supplementary Table 5), which is used to determine the electricity equivalent of heat_ccu_. E_pump_ is the electricity required to power the pumps and fans in the carbon capture unit (16.6 to 30.6 × 10^−3^ kWh/kg CO_2_, Supplementary Table 5) and E_liq_ is the electricity required to liquify the captured CO_2_ (0.089 kWh/kg CO_2_, SI Section 3 “CO_2_ Liquefaction”)).

This analysis follows the standards recommended by the National Energy Technology Laboratory (NETL)^92^ to determine the CO_2_ intensity of the electricity used to compensate for the energy penalty. NETL recommends that the energy penalty is compensated through an external electricity source, which is representative of the grid-mix of the region in which the analysis is carried out^92^. φ_Avg_ varies between 0.38 and to 0.56 kg CO_2_/kWh, which represents the lower and upper limit of the average CO_2_ intensity of electricity generated in the different grid regions in the US in 2020^92^.

Process 9—CO_2_ Transportation: This analysis assumes that the captured CO_2_ is transported in a semi-trailer truck (SI Section 3 “CO_2_ Transportation”) as it is necessary to supply the CO_2_ from the site of capture to geographically disperse concrete curing or mixing facilities, which are primarily accessible by road^21^. The CO_2_ emissions from transporting CO_2_ is the product of the total weight (φ_CCU_ plus the tare weight), the distance over which the transport occurs (D_CO2_ in km) and the CO_2_ intensity of transportation emissions of a semi-trailer truck (φ_T_ = 112 g CO_2_ per ton km, Supplementary Table 11). The transport of 1 kg of CO_2_ necessitates the transport of an additional tare weight (T_w_) of 0.4 kg in the onward trip to the CCU concrete production facility (Supplementary Table 7). In the return trip, we account for the CO_2_ emissions from the transport of only the tare weight. As a result, T_w_ equals 0.8. We assume D_CO2_ to be 810 km, which is equal to the longest distance by which CO_2_ can be transported in the U.S^93^.

Processes 10 and 11—Vaporization and injection of CO_2_: After transportation, the liquified CO_2_ needs to be vaporized to a gaseous state and injected into the concrete sample for curing or mixing^94^. The CO_2_ emissions from vaporizing (φ_Vap_) and injecting CO_2_ (φ_Inj_) is the product of φ_CCU_ (kg CO_2_), φ_Avg_ (kg CO_2_/kWh) and the electricity required to vaporize (5.3 × 10^−3^ kWh/kg CO_2_, SI Section 3) and inject CO_2_ (37 × 10^−3^ kWh/kg CO_2_)^16^, respectively. η is the CO_2_ absorption efficiency and represents the portion of the total CO_2_ which is absorbed during mixing or curing of concrete (datasets 71 to 99). η varies between 50% and 85% during mixing^16,19,52^. For curing, η is equal to 1 (i.e.,100% absorption) as the curing datasets (datasets 1 to 70) report CO_2_ utilized as the ratio of the mass of CO_2_ absorbed to the mass of cement.

Processes 12 and 13—CO_2_ and steam curing: The CO_2_ emissions from CO_2_ curing of the concrete sample (φ_CO2_Cur_) is the product of φ_CCU_ (kg CO_2_), φ_Avg_ (kg CO_2_/kWh), the electrical power requirements of the curing chamber (P_CO2_Cur_ = 38.8 kW/m^3^ of concrete)^35,95^ and the duration of curing (t_CO2_Cur_ in hours, SI Section 2), which is determined from the literature review^38,96^. φ_CO2_Cur_ is normalized to the compressive strength of the concrete sample. In some datasets, a combination of steam and CO_2_ curing is used for the production of CCU concrete. In this case, the analysis includes the CO_2_ emissions from steam curing of CCU concrete. The CO_2_ emissions from steam curing (φ_Stm_Cur_) is the product of CO_2_ intensity of steam curing (39.55 kg CO_2_/m^3^/h, Supplementary Table 8) and the duration of steam curing (t_stm_Cur_ in hours), which is determined from the literature (Supplementary Table 1 Process 13). φ_Stm_Cur_ is normalized to the compressive strength of the concrete sample.

When CO_2_ is used for mixing of concrete (datasets in category 3 and 4), the CO_2_ emissions from CO_2_ and steam curing are assumed to be zero as CO_2_ curing of concrete is not conducted.

### Conventional concrete production CO_2_ emissions

The total life cycle CO_2_ emissions from producing conventional concrete (TOT_Conv_) are similarly quantified in Eq. 5.

5 $${\mathrm{TOT}}_{{\mathrm{Conv}}} = \, {\mathrm{(}}\upvarphi _{\mathrm{C}} \ast {\mathrm{C}}_{{\mathrm{Conv}}}{\mathrm{)}} + {\mathrm{(}}\upvarphi _{{\mathrm{CA}}} \ast {\mathrm{CA}}_{{\mathrm{conv}}}{\mathrm{)}} + {\mathrm{(}}\upvarphi _{{\mathrm{FA}}} \ast {\mathrm{FA}}_{{\mathrm{conv}}}{\mathrm{)}} + {\mathrm{(}}\upvarphi _{\mathrm{W}} \ast {\mathrm{W}}_{{\mathrm{conv}}}{\mathrm{)}} \\ \,+{\mathrm{(}}\upvarphi _{{\mathrm{SCM}}} \ast {\mathrm{SCM}}_{{\mathrm{conv}}}{\mathrm{)}} + {\mathrm{(E}}_{\mathrm{p}} \ast \upvarphi _{{\mathrm{Pow}}\_{\mathrm{Plnt}}} \ast {\mathrm{Alloc}}_{{\mathrm{elec}}}{\mathrm{)}} + \upvarphi _{{\mathrm{Stm}}\_{\mathrm{Cur}}} + {\mathrm{(D}}_{\mathrm{M}} \ast \upvarphi _{{\mathrm{TM}}} \ast {\mathrm{M}}_{{\mathrm{Conv}}}{\mathrm{)}}$$

(Ep * φPow_Plnt* Allocelec) quantifies the CO_2_ emissions from generating E_p_ kWh of electricity in a power plant without carbon capture. φ_Pow_Plnt_ is the CO_2_ intensity of electricity generated in a coal or NGCC plant (kg CO_2_/kWh, SI Supplementary Table 1).

### Net CO_2_ benefit and sensitivity analysis

The difference between the TOT_CCU_ (Eq. 1) and TOT_Conv_ (Eq. 5) determines the net CO_2_ benefit from CCU concrete substituting conventional concrete.

6 $${\mathrm{Net}}\;{\mathrm{CO}}_{\mathrm{2}}{\mathrm{Benefit}} = {\mathrm{TOT}}_{{\mathrm{Conv}}}{\mathrm{ - TOT}}_{{\mathrm{CCU}}}$$

TOT_CCU_ and TOT_Conv_ are driven by the CO_2_ emissions from the 13 processes, which, in turn, are impacted by the uncertainty and variability in the underlying parameters (Supplementary Table 1).

In the scatter plot analysis, 10,000 values are stochastically generated for the material and inventory items and the parameters for the 13 processes, which are obtained from the dataset (ranges and relationships presented in Supplementary Table 1). The stochastically generated values are applied in Eqs. 1, 5, and 6 to determine the CO_2_ emissions from the 13 processes for conventional and CCU concrete and the net CO_2_ benefit. The net CO_2_ benefit is plotted on the ordinate. The difference between the CO_2_ emissions for each of the 13 contributing processes in conventional and concrete is plotted on the abscissa.

To further verify the results, this analysis conducts a moment independent sensitivity analysis^25,29,30,97^ to determine the process (from the 13 processes) having the most impact on the net CO_2_ benefit. The moment independent sensitivity analysis determines the δ index for each of the 13 processes. The δ index quantifies the relative contribution of each of the 13 processes to the probability distribution function of the net CO_2_ benefit. The moment independent sensitivity analysis offers methodological advantages as it accounts for the correlation between the input parameters for the 13 processes and is applicable when the input parameters and the output are not linearly related^98^. This study determines the δ indices over 10,000 Monte Carlo runs based on the approach presented in Wei, Lu, and Yuan^97^.

## Supplementary information

Supplementary Information

Peer Review File

## Data Availability

All the datasets utilized and analyzed in this study are included in section 2 of the Supplementary information file.

## Code availability

The code used in the analysis is included in section 8 of the Supplementary information file. The code can be accessed on ref. ^99^.
