# Supplementary material for: Carbon dioxide utilization in concrete curing or mixing might not produce a net climate benefit
Source: Nat Commun. 2021 Feb 8;12:855. doi: 10.1038/s41467-021-21148-w (PMC7870952; doi:10.1038/s41467-021-21148-w)
Supplement: Supplementary file 1 — Supplementary Information [file 41467_2021_21148_MOESM1_ESM.pdf]

Supplementary Information

To accompany the manuscript titled

# Carbon dioxide utilization in concrete curing or mixing might not produce a net climate benefit


Dwarakanath Ravikumar[1,4*], Duo Zhang[2], Gregory Keoleian[1], Shelie Miller[1], Volker Sick[3], Victor Li[2]

1. Center for Sustainable Systems (CSS), School for Environment and Sustainability (SEAS), University of Michigan
2. Department of Civil and Environmental Engineering, University of Michigan
3. Department of Mechanical Engineering, University of Michigan
4. National Renewable Energy Laboratory (NREL), USA

*Corresponding Author, email: dwarak.ravikumar@nrel.gov


Comprising:

46 Pages

12 Tables

14 Figures

## 1. Expression and range of the 13 process parameters used to quantify the $CO_2$ impact of producing CCU and conventional concrete

**Supplementary Table 1 Expression and range of the 13 process parameters used to quantify the $CO_2$ emissions from producing CCU and conventional concrete**

| Process | CCU concrete production: Expression and range of the parameters used to quantify the $CO_2$ impact of the process | Conventional concrete production: Expression and range of the parameters used to quantify the $CO_2$ impact of the process |
|---|---|---|
| Process 1: OPC production | ($\varphi_C$ * $C_{CCU}$)<br>• $\varphi_C$: Cement $CO_2$ intensity. $\mu$ = 0.948, $\sigma$ = 0.15 kg $CO_2$/kg cement (supplementary Information (SI) Supplementary Table 2)<br>• $C_{CCU}$: Mass of cement used in producing CCU concrete (kg/m$^3$/MPa) which is determined from literature review (SI Section 2) | ($\varphi_C$ * $C_{Conv}$)<br>• $\varphi_C$: Cement $CO_2$ intensity. $\mu$ = 0.948, $\sigma$ = 0.15 kg $CO_2$/kg cement (Supplementary Table 2)<br>• $C_{Conv}$: Mass of cement used in producing conventional concrete (kg/m$^3$/MPa) which is determined from literature review (SI Section 2) |
| Process 2: Coarse aggregate production | ($\varphi_{CA}$ * $CA_{CCU}$)<br>• $\varphi_{CA}$: Coarse aggregate $CO_2$ intensity. $\mu$ = 5x10$^{-3}$, $\sigma$ = 1x10$^{-3}$kg $CO_2$/kg coarse aggregate (Supplementary Table 2)<br>• $CA_{CCU}$: Mass of coarse aggregate used in producing CCU concrete (kg/m$^3$/MPa) which is determined from literature review (SI Section 2) | ($\varphi_{CA}$ * $CA_{Conv}$)<br>• $\varphi_{CA}$: Coarse aggregate $CO_2$ intensity. $\mu$ = 5x10$^{-3}$, $\sigma$ = 1x10$^{-3}$kg $CO_2$/kg coarse aggregate (Supplementary Table 2)<br>• $CA_{Conv}$: Mass of coarse aggregate used in producing conventional concrete (kg/m$^3$/MPa) which is determined from literature review (SI Section 2) |
| Process 3: Fine aggregate production | ($\varphi_{FA}$ * $FA_{CCU}$)<br>• $\varphi_{FA}$: Fine aggregate $CO_2$ intensity. $\mu$ = 4x10$^{-3}$, $\sigma$ = 4x10$^{-4}$kg $CO_2$/kg fine aggregate (Supplementary Table 2)<br>• $FA_{CCU}$: Mass of fine aggregate used in producing CCU concrete (kg/m$^3$/MPa) which is determined from literature review (Section 2 SI) | ($\varphi_{FA}$ * $FA_{Conv}$)<br>• $\varphi_{FA}$: Fine aggregate $CO_2$ intensity. $\mu$ = 4x10$^{-3}$, $\sigma$ = 4x10$^{-4}$kg $CO_2$/kg fine aggregate (Supplementary Table 2)<br>• $FA_{Conv}$: Mass of fine aggregate used in producing conventional concrete (kg/m$^3$/MPa) which is determined from literature review (Section 2 SI) |
| Process 4: Water production | ($\varphi_W$ * $W_{CCU}$)<br>• $\varphi_W$: Water $CO_2$ intensity. $\mu$ = 1x10$^{-3}$, $\sigma$ = 2x10$^{-4}$kg $CO_2$/kg water (Supplementary Table 2)<br>• $W_{CCU}$: Mass of water used in producing CCU concrete (kg/m$^3$/MPa) which is determined from literature review (Section 2 SI) | ($\varphi_W$ * $W_{Conv}$)<br>• $\varphi_W$: Water $CO_2$ intensity. $\mu$ = 1x10$^{-3}$, $\sigma$ = 2x10$^{-4}$kg $CO_2$/kg water (Supplementary Table 2)<br>• $W_{Conv}$: Mass of water used in producing conventional concrete (kg/m$^3$/MPa) which is determined from literature review (Section 2 SI) |
| Process 5: SCM production | ($\varphi_{SCM}$ * $SCM_{CCU}$)<br>• $\varphi_{SCM}$: SCM $CO_2$ intensity (kg $CO_2$/m$^3$), refer SI sections 4, 5, or 6 depending on whether system boundary expansion or economic value or mass based allocation is used, respectively.<br>• $SCM_{CCU}$: Mass of SCM used in producing CCU concrete (kg/m$^3$/MPa) which is | ($\varphi_{SCM}$ * $SCM_{Conv}$)<br>• $\varphi_{SCM}$: SCM $CO_2$ intensity (kg $CO_2$/m$^3$), refer SI sections 4, 5, or 6 depending on whether system boundary expansion or economic value or mass based allocation is used, respectively. |

| Process | CCU concrete production: Expression and range of the parameters used to quantify the $CO_2$ impact of the process | Conventional concrete production: Expression and range of the parameters used to quantify the $CO_2$ impact of the process |
|---|---|---|
| | determined from literature review (Section 2 SI) | • $SCM_{Conv}$: Mass of SCM used in producing conventional concrete (kg/m$^3$/MPa) which is determined from literature review (Section 2 SI) |
| Process 6: Transporting materials | ($D_M$*$\varphi_T$* $M_{CCU}$)<br>• $D_M$: Distance (km) over which materials required to produce CCU concrete is transported. $D_M$ is assumed to be equal to the national average transportation distance in the US concrete industry (SI Section 7 Supplementary Table 10)<br>• $\varphi_T$: $CO_2$ emissions from transportation via road, rail, ocean and barge (kg $CO_2$ per tonne-km). Refer SI Section 7 Supplementary Table 11.<br>• $M_{CCU}$: Mass of $C_{CCU}$, $CA_{CCU}$, $FA_{CCU}$, $W_{CCU}$ and $SCM_{CCU}$ required to manufacture CCU concrete, which is determined form the literature review (SI Section 2) | ($D_M$*$\varphi_T$* $M_{Conv}$)<br>• $M_{Conv}$: Mass of $C_{Conv}$, $CA_{Conv}$, $FA_{Conv}$, $W_{Conv}$ and $SCM_{Conv}$ required to manufacture CCU concrete, which is determined form the literature review (SI Section 2) |
| Process 7: MEA system production | ($\varphi_{CCU}$ * $\varphi_{MEA}$)<br>• $\varphi_{CCU}$: Mass of $CO_2$ captured (kg) which is determined from literature review (SI Section 2)<br>$\varphi_{MEA}$: Life cycle $CO_2$ emissions from producing MEA $CO_2$ capture system (kg $CO_2$/kg $CO_2$ captured). Lognormal distribution: $\mu$= 6x10$^{-3}$, $\sigma$ = 2x10$^{-3}$ kg $CO_2$/kg $CO_2$ captured (SI Section 3) | Zero. $CO_2$ is not captured when conventional concrete is produced. |
| Process 8: Total power plant emissions | ($Alloc_{elec}$ * $\varphi_{Not\ Cap}$) + ($\varphi_{Avg}$ * $E_p$)<br>• $Alloc_{elec}$ quantifies the allocation of $CO_2$ emissions from a coal power plant between the co-products of electricity and fly ash, which is used as SCM in concrete production in certain datasets.<br>$Alloc_{elec}$ is 0.98 or 0.94 as economic or mass allocation allocates 0.02 and 0.06 of the total $CO_2$ emissions from the coal power plant to the co-product of fly ash (SI Sections 5 and 6). $Alloc_{elec}$ is 1when $CO_2$ is captured from a natural gas combined cycle power plant or when system boundary expansion is used (instead of economic or mass allocation).<br>• $\varphi_{Not\ Cap}$ = (1/9) * $\varphi_{CCU}$. 90% of the emitted $CO_2$ is captured and utilized ($\varphi_{CCU}$) and 10% of the $CO_2$ is not captured ($\varphi_{Not\ Cap}$).<br>• $\varphi_{Avg}$: Electricity $CO_2$ intensity 0.386 to 0.568 kg $CO_2$/kWh, which is the average life cycle $CO_2$ intensity of electricity in the different grid regions in 2020 in the U.S (Exhibit 2-15 in [1])<br>• $E_p$ = $\varphi_{CCU}$*($E_{pump}$+(heat*hte*0.277)+$E_{Liq}$)<br>• $\varphi_{CCU}$: Mass of $CO_2$ captured (kg) which is determined from literature review (SI Section 2) | ($E_p$ * $\varphi_{Pow\_Plnt}$ * $Alloc_{elec}$)<br>When $CO_2$ is captured from a coal power plant<br>• $\varphi_{Pow\_Plnt}$: Electricity $CO_2$ intensity of electricity generated from a coal plant (kg $CO_2$/kWh). $\varphi_{Pow\_Plnt}$ is determined in SimaPro using the ecoinvent dataset "Electricity, high voltage WECC, US Only electricity production, hard coal Alloc Def, U". $\varphi_{Pow\_Plnt}$ is lognormally distributed and has a mean of 1.24 and standard deviation of 0.039 kg $CO_2$/kWh.<br><br>When $CO_2$ is captured from a NGCC power plant<br>• $\varphi_{Pow\_Plnt}$: Electricity $CO_2$ intensity of electricity generated from a combined cycle natural gas (NGCC) plant (kg $CO_2$/kWh). $\varphi_{Pow\_Plnt}$ is determined in SimaPro using the ecoinvent dataset "Electricity, high voltage CA-SK electricity production, natural gas, combined cycle power plant Alloc Def, U". $\varphi_{Pow\_Plnt}$ is lognormally distributed |

| Process | CCU concrete production: Expression and range of the parameters used to quantify the $CO_2$ impact of the process | Conventional concrete production: Expression and range of the parameters used to quantify the $CO_2$ impact of the process |
|---|---|---|
| | • $E_{pump}$: Electricity to operate pump and fans in $CO_2$ capture unit: 16.6 to 30.6 kWh/ton $CO_2$ captured for coal power plants and 58.3 to 91.6 kWh/ton $CO_2$ captured for natural gas combined cycle power plants (SI Section 3)<br>• heat: Heat used for $CO_2$ capture: 2700-3300 MJ/ton $CO_2$ captured for coal plants and 2900 MJ/ton $CO_2$ for natural gas combined cycle plants (SI Section 3)<br>• hte: Heat to electricity ratio: 0.09 to 0.25 (SI Section 3)<br>• $E_{Liq}$: Electricity required for $CO_2$ liquefaction. 89 kWh/ton $CO_2$ (SI Section 3). | and has a mean of 0.46 and standard deviation of 0.037 kg $CO_2$/kWh. |
| Process 9: $CO_2$ Transportation | ($\varphi_{CCU}$ * (1+2$T_w$) * $D_{CO2}$ *$\varphi_T$)<br>• $\varphi_{CCU}$: Mass of $CO_2$ captured (kg) which is determined from literature review (SI Section 2)<br>• $T_W$ is the additional 0.4 kg of tare weight of the semi-trailer when 1 kg of $CO_2$ is transported to the CCU concrete production facility (SI Section 3, "$CO_2$ Transportation"). 2$T_W$ accounts for the transport of the 0.4 kg of tare weight in the onward and the return trip.<br>• $D_{CO2}$: Distance (km) over which $CO_2$ is transported to be used in CCU concrete production. $D_{CO2}$ is assumed to be equal to 810 km, which is equal to the longest distance by which $CO_2$ can be transported in the U.S.[2]<br>• $\varphi_T$: $CO_2$ emissions from transportation in a semi-trailer truck: 0.112 kg $CO_2$/kg-km (SI Section 3 "$CO_2$ Transport" and Supplementary Table 11) | Zero. $CO_2$ is not captured and transported when conventional concrete is produced. |
| Process 10: $CO_2$ Vaporization | ($\varphi_{CCU}$ * $\varphi_{Vap}$)<br>• $\varphi_{CCU}$: Mass of $CO_2$ captured (kg) which is determined from literature review (SI Section 2)<br>• $\varphi_{Vap}$= $E_{vap}$* $\varphi_{Avg}$.<br>• $E_{vap}$ = 5.3 kWh/ton $CO_2$, which is the electricity required for vaporizing $CO_2$ (SI Section 3 "$CO_2$ Vaporization"<br>• Supplementary Table **8**).<br>• $\varphi_{Avg}$: Electricity $CO_2$ intensity 0.386 to 0.568 kg $CO_2$/kWh (average values for the different grid regions in the US[1]) | Zero. $CO_2$ is not vaporized when conventional concrete is produced. |
| Process 11: $CO_2$ Injection | ($\varphi_{CCU}$ * $\varphi_{Inj}$) + ((1-η) * $\varphi_{CCU}$)<br>• $\varphi_{CCU}$ is determined from literature review (SI Section 2)<br>• $\varphi_{Inj}$= $E_{Inj}$* $\varphi_{Avg}$.<br>• $E_{Inj}$ = 37 kWh/ton $CO_2$, which is the electricity required for injecting $CO_2$.[3]<br>• $\varphi_{Avg}$: Electricity $CO_2$ intensity 0.386 to 0.568 kg $CO_2$/kWh (average values for the different grid regions in the US[1]) | Zero. $CO_2$ is not injected when conventional concrete is produced. |

| Process | CCU concrete production: Expression and range of the parameters used to quantify the $CO_2$ impact of the process | Conventional concrete production: Expression and range of the parameters used to quantify the $CO_2$ impact of the process |
|---|---|---|
| | • $\eta$ is the $CO_2$ absorption efficiency and represents the portion of the total $CO_2$ which is absorbed during mixing of concrete (datasets 71 to 99). $\eta$ varies between 50% to 85% during mixing.[3-5] For curing datasets (datasets 1 to 70), $\eta$ is assumed to be equal to 1 (i.e.100% absorption). | |
| Process 12: $CO_2$ curing | $\varphi_{CO2\_Cur} = (\varphi_{Avg} * P_{CO2\_Cur} * t_{CO2\_Cur})/Strength_{CCU}$<br>• $\varphi_{Avg}$: Electricity $CO_2$ intensity 0.386 to 0.568 kg $CO_2$/kWh (average values of the different grid regions in the US[1])<br>• $P_{CO2\_Cur}$ = 38.8 kW/$m^3$, which is the power required for $CO_2$ curing. (SI Section 3 Supplementary Table 9)<br>• $t_{CO2\_Cur}$ = number of hours of $CO_2$ curing of CCU concrete, which is determined from the literature review (SI Section 2)<br>• $Strength_{CCU}$: compressive strength of CCU concrete, which is determined from the literature review (SI Section 2) | Zero. $CO_2$ curing is not used when conventional concrete is produced. |
| Process 13: Steam curing | $\varphi_{Stm\_Cur} = (CO_{2\_Stm\_Cur} * t_{steam\_Cur})/Strength_{CCU}$<br>• $CO_{2\_Stm\_Cur}$: 39.55 kg $CO_2$/$m^3$/hour, which is the hourly $CO_2$ emissions from steam curing (SI Section 3 Supplementary Table 9)<br>• $t_{steam\_Cur}$: number of hours of steam curing of CCU concrete, which is determined from the literature review (SI Section 2)<br>• $Strength_{CCU}$: compressive strength of CCU concrete, which is determined from the literature review (SI Section 2) | $\varphi_{Stm\_Cur} = (CO_{2\_Stm\_Cur} * t_{steam\_Cur})/Strength_{Conv}$<br>• $\varphi_{Steam\_Cur:}$ 39.55 kg $CO_2$/$m^3$/hour of steam curing (SI Section 3 Supplementary Table 9)<br>• $t_{steam\_Cur}$: number of hours of steam curing of conventional concrete, which is determined from literature review (SI Section 2)<br>• $Strength_{Conv}$: compressive strength of conventional concrete, which is determined from literature review (SI Section 2) |

## Supplementary Table 2 Ecoinvent datasets used in SimaPro to determine the life cycle $CO_2$ emissions from producing inventory items required for concrete production

| Inventory item used in concrete | Ecoinvent dataset used in SimaPro to determine the $CO_2$ emission from producing the inventory item | Mean ($\mu$) and standard deviation ($\sigma$) of life cycle $CO_2$ emission from producing 1 kg of inventory item (kg $CO_2$/kg inventory item) |
|---|---|---|
| Cement | Cement, Portland US\| production \| Alloc Def, U | $\mu = 0.948$, $\sigma = 0.15$ |
| Coarse aggregate | Gravel, crushed CH\| production \| Alloc Def, U | $\mu = 5x10^{-3}$, $\sigma = 1x10^{-3}$ |
| Fine aggregate | Sand RoW\| gravel and quarry operation \| Alloc Def, U | $\mu = 4x10^{-3}$, $\sigma = 4x10^{-4}$ |
| Water | Water, deionised, from tap water, at user RoW\| production \| Alloc Def, U | $\mu = 1x10^{-3}$, $\sigma = 2x10^{-4}$ |

## 2. Literature Review to Determine Inventory Requirement for Conventional and CCU Concrete in each of the 99 datasets

The four tabs in the excel (accessible at the below link) contains parameters from the 99 datasets, which were obtained from a review of published studies comparing conventional concrete and CCU concrete i.e. concrete produced through $CO_2$ mixing and $CO_2$ curing.

The parameters in the red column - compressive strength ($Strength_{Conv}$) and steam curing hours ($t_{Stm\_Cur}$) - are specific to conventional concrete.

The parameters in the green column - $CO_2$ utilized ($\varphi_{CCU}$), compressive strength ($Strength_{CCU}$), carbon curing hours ($t_{CO2\_Cur}$) and steam curing hours ($t_{Stm\_Cur}$) - are specific to conventional concrete.

The parameters in the grey columns - cement, coarse aggregate, fine aggregate, water and SCM used - are common across conventional and CCU concrete production.

https://www.dropbox.com/s/ueex0uk3m95q3eo/Literature.xlsx?dl=0

**Supplementary Table 3 Summary of the excel sheet containing 99 datasets, which were obtained from the literature review and organized into 4 categories**

| Excel Tab | Dataset Category | Captured $CO_2$ used for? | SCM used along with OPC? | Number of datasets in category |
|---|---|---|---|---|
| 1 | 1 | Curing | No | 50 |
| 2 | 2 | Curing | Yes | 20 |
| 3 | 3 | Mixing | No | 8 |
| 4 | 4 | Mixing | Yes | 21 |
| | | | Total | 99 |

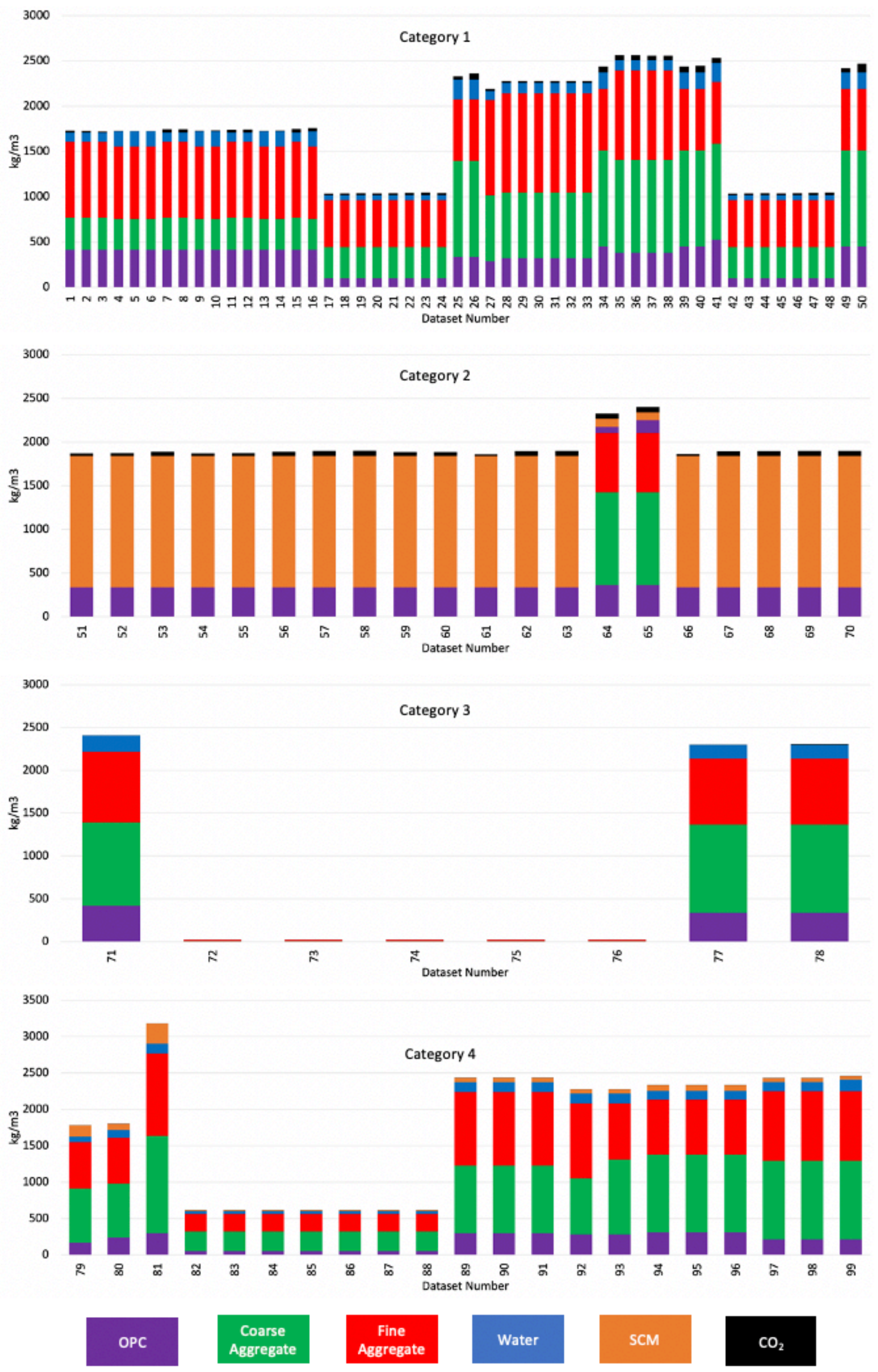


**Supplementary Figure 1 Summary of the materials used in the design mix across the 99 datasets. $CO_2$ is used only in the production of CCU concrete. All the values are in kg/m$^3$ of concrete.**

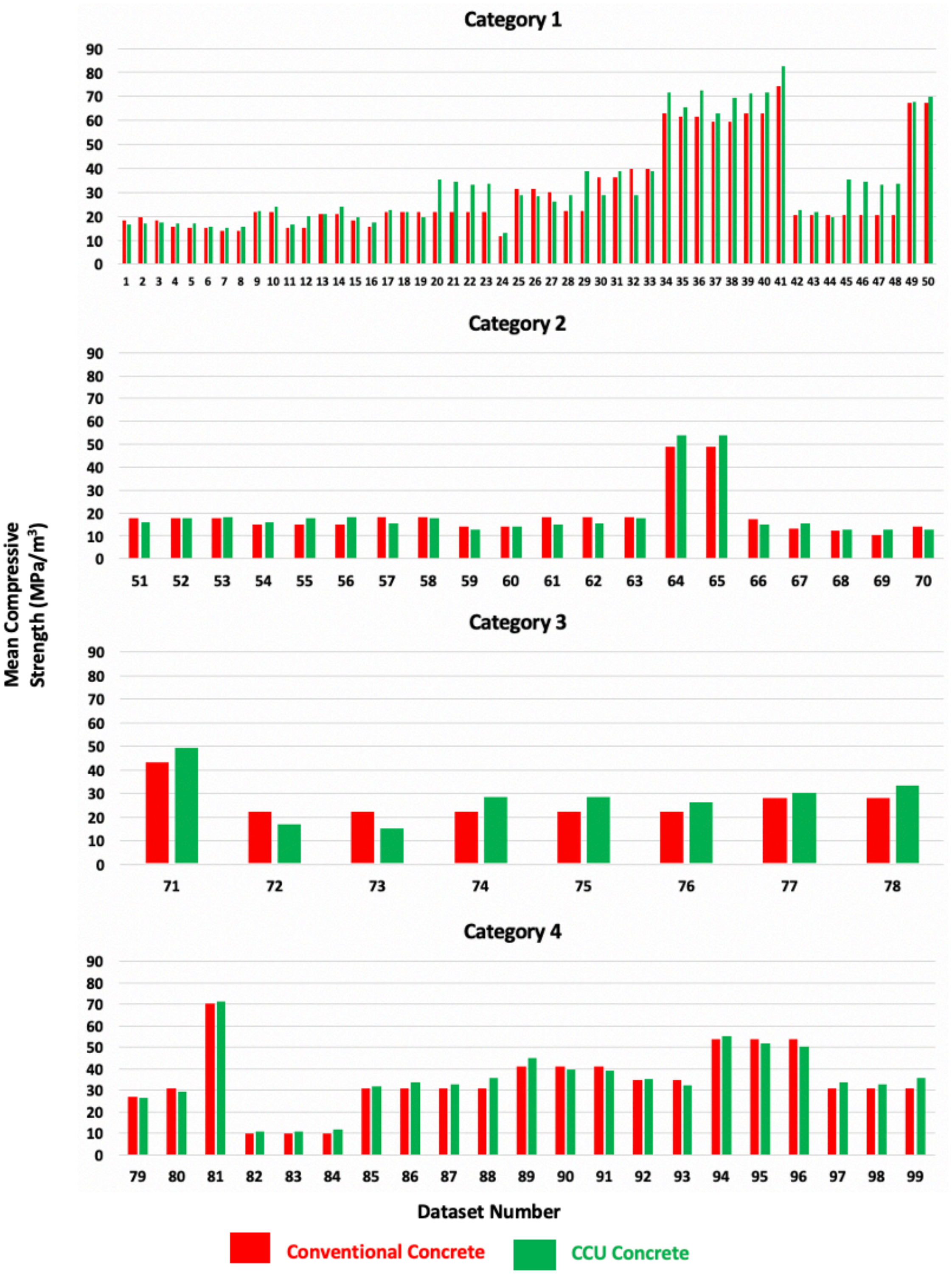


**Supplementary Figure 2 Compressive strength of conventional and CCU concrete across the 99 datasets.**

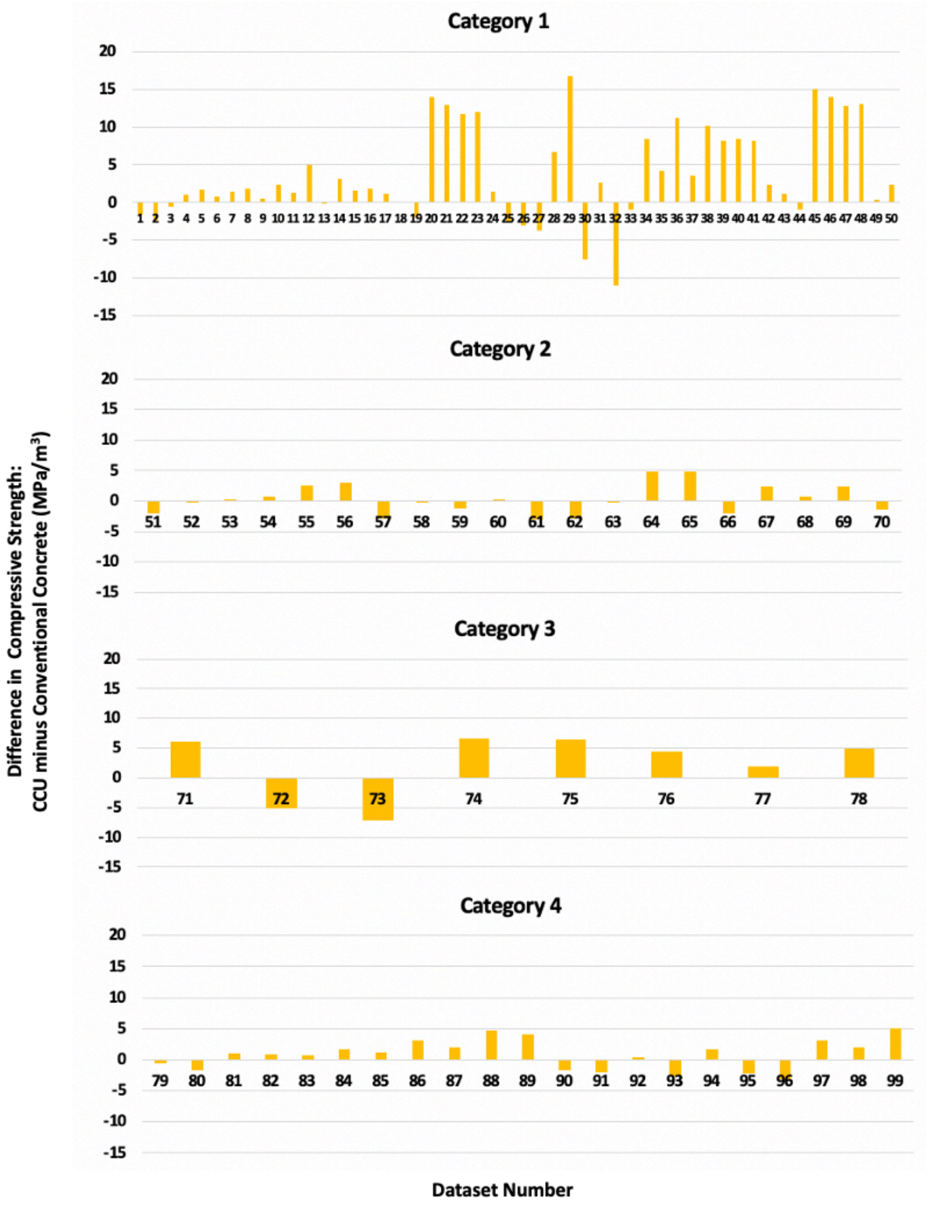


**Supplementary Figure 3 Difference between the compressive strength of conventional and CCU concrete across the 99 datasets. If the difference is negative, then the compressive strength of CCU concrete is lower than conventional concrete. If the difference is positive, then the compressive strength of CCU concrete is greater than conventional concrete. The strength of CCU concrete is lower than conventional concrete in 31 datasets.**

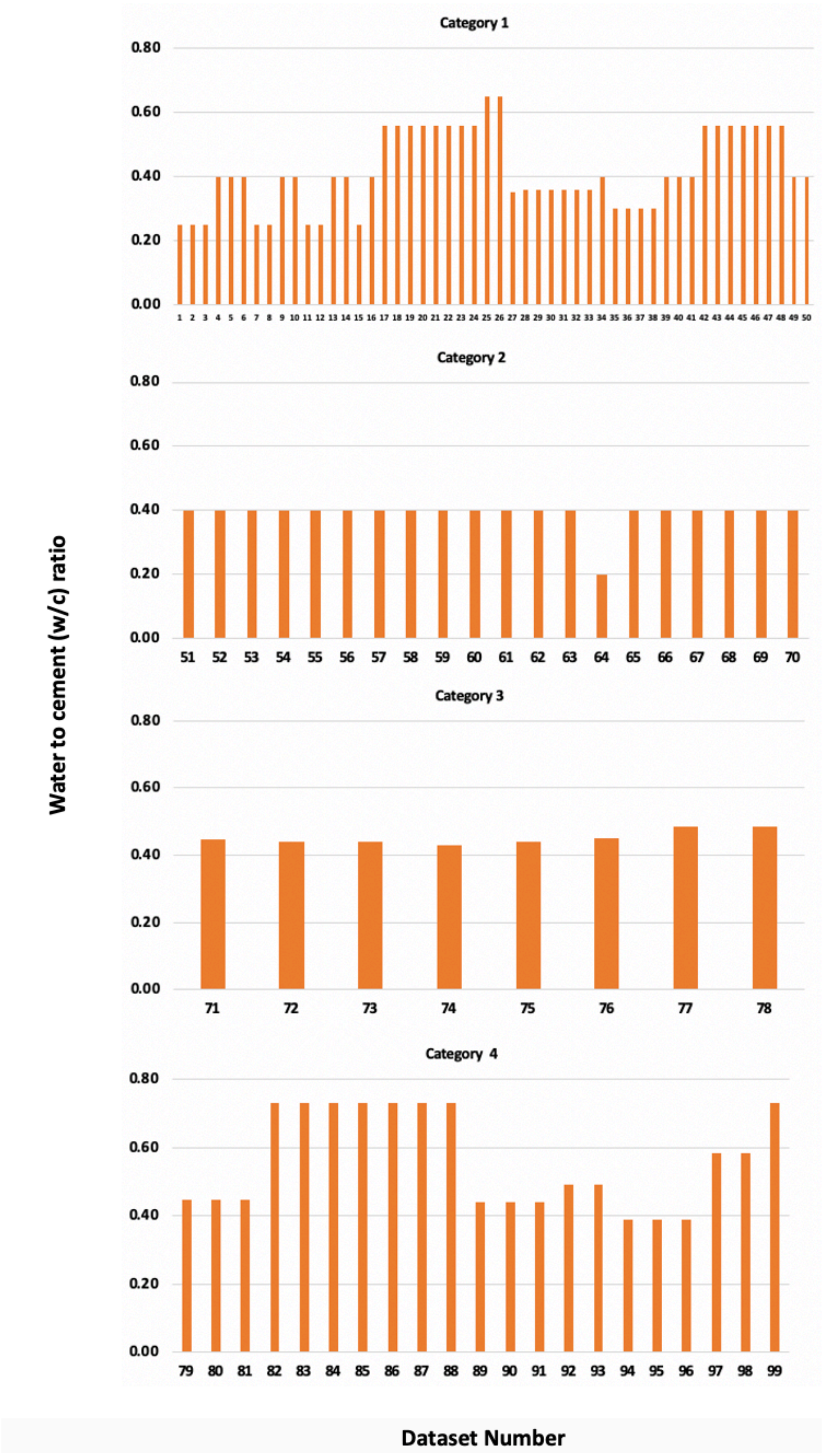


**Supplementary Figure 4 Water to cement (w/c) ratio across the 99 datasets**

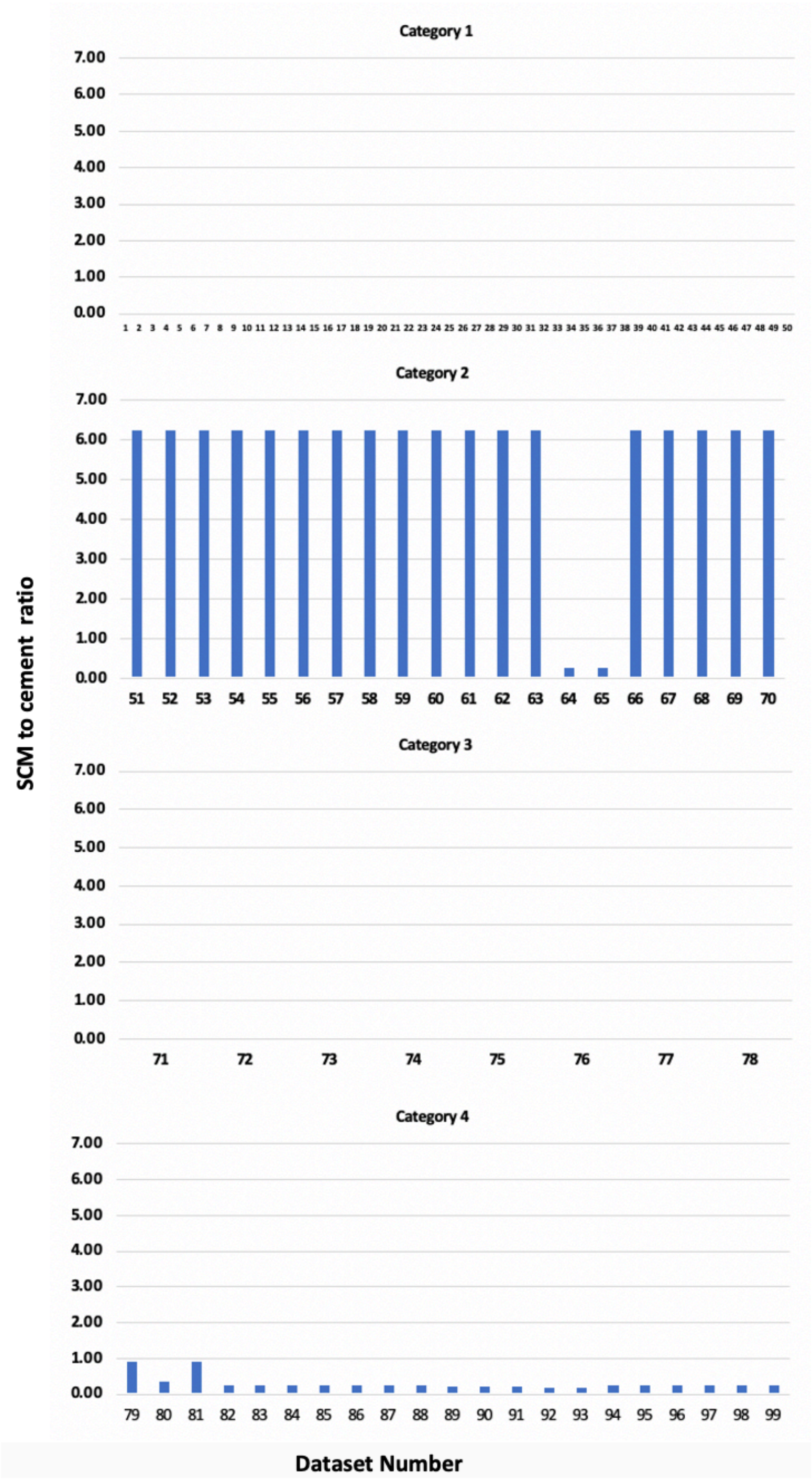


**Supplementary Figure 5 SCM to cement ratio across the 99 datasets**

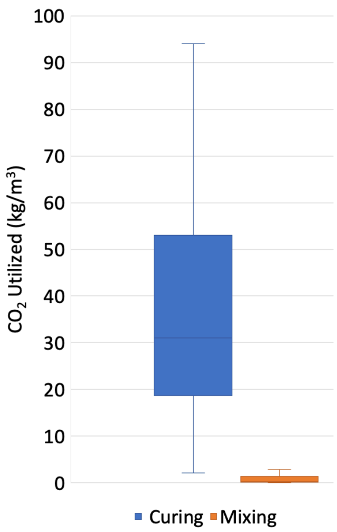


**Supplementary Figure 6 Summary of the mass of $CO_2$ utilized per $m^3$ of concrete in curing (Datasets 1 to 70) and mixing datasets (Datasets 71 to 99).**

## 3. Inventory Requirement to Capture, Liquify, Transport and Vaporize $CO_2$

This study obtained the material requirements for post combustion MEA-based capture of $CO_2$ based on a literature review of 21 studies. The excel containing the details of the literature review is available in the link below

https://www.dropbox.com/s/nce6bqle16xup35/Carbon%20Capture%20MEA.xlsx?dl=0

The results and the Ecoinvent dataset used to determine the life cycle $CO_2$ emissions of the material inventory items of a post-combustion MEA system are summarized in Supplementary Table 4.

By characterizing the Ecoinvent datasets using the global warming mid-point impact category in the TRACI[6] impact assessment method in SimaPro, we obtain a value mean of 50 and standard deviation 3.91 kg of $CO_2$ emissions per 1000 kg of $CO_2$ captured using post-combustion MEA system.

**Supplementary Table 4 Material inventory requirements for post-combustion capture of 1000 kg of $CO_2$ using MEA.**

| Material inventory item | Average Value (per ton $CO_2$) | Lower limit (per ton $CO_2$) | Upper limit (per ton $CO_2$) | Scenario specific ecoinvent dataset used to determine the $CO_2$ impact |
|---|---|---|---|---|
| MEA input(kg) | 1.65 | 0.2 | 3.1 | Monoethanolamine \| ethanolamine production \| Alloc Def, U |
| Activated carbon (kg) | 0.059 | 0.037 | 0.082 | Activated carbon, at plant/RER Mass |
| NaOH (kg) | 0.1565 | 0.013 | 0.3 | Sodium hydroxide, without water, in 50% solution state \| chlor-alkali electrolysis, membrane cell \| Alloc Def, U |
| Ammonia emissions (kg) | 0.17 | 0.035 | 0.315 | Ammonia - Standard dataset available in SimaPro in the Emissions to air category |
| MEA emissions (kg) | 3.1E-02 | 6.27E-05 | 0.063 | Monoethanolamine - Standard dataset available in SimaPro in the Emissions to air category |
| Water (kg) | 655 | 210 | 1100 | Water, deionised, from tap water, at user \| production \| Alloc Def, U |
| Formaldehyde emissions (kg) | 1.31E-04 | 2.62E-07 | 2.62E-04 | Formaldehyde - Standard dataset available in SimaPro in the Emissions to air category |
| Acetaldehyde emissions (kg) | 8.35E-05 | 1.67E-07 | 1.67E-04 | Acetaldehyde - Standard dataset available in SimaPro in the Emissions to air category |

Electricity is required in the carbon capture facility to power the pump necessary for transporting the solvent through the absorber unit and the fans which help overcome the pressure drop in the absorber unit.[7]

The analysis includes the heat required for $CO_2$ capture, which is necessary to reclaim and regenerate the amine[8] and is determined through a literature review. The heat used for $CO_2$

capture can instead be used to generate electricity in a scenario without carbon capture. As a result, to quantify the electricity loss from the heat used for $CO_2$ capture, we determine the product of the heat used for $CO_2$ capture and the heat to electricity ratio.

The total electricity requirement for $CO_2$ capture is the sum of the electricity to operate the fans and pumps, electricity is required to $CO_2$ compression and the electricity equivalent of heat used in the $CO_2$ capture process and is summarized in Supplementary Table 5.

**Supplementary Table 5 Energy requirements for post-combustion capture and liquefaction of 1 ton of $CO_2$ using MEA in coal and natural gas combined cycle power plants.**

| Power plant | Energy inventory item for carbon capture using MEA | Average value | Lower limit | Upper limit | Range of values reported in literature |
|---|---|---|---|---|---|
| Coal | Heat energy for MEA regeneration | 3000 MJ/ton $CO_2$ | 2700 MJ/ton $CO_2$ | 3300 MJ/ton $CO_2$ | 2700 to 3300 MJ/ton, page 117[9]<br>2900 MJ/ton[10,11]<br>2700 MJ/ton[12] |
| | Electricity to operate pump and fans in $CO_2$ capture unit | 23.6 kWh/ton $CO_2$ | 16.6 kWh/ton $CO_2$ | 30.6 kWh/ton $CO_2$ | 23.6 kWh/ton $CO_2$ – Table 3[7], 0.06 to 0.11 GJ electricity/ton $CO_2$ - page 117[13] |
| | Electricity required to liquify $CO_2$ to 20 bars | 89 kWh/ton $CO_2$ | | | Refer “$CO_2$ Liquefaction” section below |
| Natural gas combined cycle (NGCC) | Heat energy for MEA regeneration | 2900 MJ/ton $CO_2$ | 2900 MJ/ton $CO_2$ | 2900 MJ/ton $CO_2$ | 2900 MJ/ton[12] |
| | Electricity to operate pump and fans in $CO_2$ capture unit | 75 kWh/ton $CO_2$ | 58.3 kWh/ton $CO_2$ | 91.6 kWh/ton $CO_2$ | 0.21 to 0.33 GJ electricity/ton $CO_2$ - page 117[13] |
| | Electricity required to liquify $CO_2$ to 20 bars | 89 kWh/ton $CO_2$ | | | Refer “$CO_2$ Liquefaction” section below |
| Coal and NGCC | Heat to electricity ratio (hte) | 0.17 | 0.09 | 0.25 | 0.2 – Table 8[14]<br>0.14 to 0.25 – Range of values, which [15] reports based on a literature review.<br>0.09 to 0.25 – Range of values, which [16] reports based on a literature review. |

## $CO_2$ Liquefaction

We assume the $CO_2$ liquification is conducted through a closed refrigeration system using ammonia as the refrigerant, which is widely used.[17] The electricity required for liquefaction of $CO_2$ are summarized in Supplementary Table 6.

**Supplementary Table 6 Electricity required for $CO_2$ liquefaction.**

| Input stream $CO_2$ pressure (bar) | Output liquid $CO_2$ pressure (bar) | Electricity requirement (kWh/ton) | Reference |
|---|---|---|---|
| 1 | 7 | 98 | [18] |
| 1 | 10 | 94 | [18] |
| 1 | 15 | 90 | [18] |
| 1 | 20 | 89 | [18] |
| 1 | 25 | 87 | [18] |
| 1 | 30 | 86 | [18] |
| 1 | 40 | 85 | [18] |
| 1 | 50 | 86 | [18] |
| 1 | 60 | 88 | [18] |
| 1 | 70 | 90 | [18] |
| 1.1 | 6.5 | 110 | [17] |
| 1.8 | 45 | 87 | [19] |
| 1.8 | 55 | 83 | [19] |
| 1.8 | 65 | 85 | [19] |
| 1 | 6.5 | 105 | [20] |
| 1 | 81 | 99 | [21] |
| 1 | 81 | 79 | [21] |
| 1 | 81 | 78 | [21] |
| 1 | 81 | 76 | [21] |
| 1 | 81 | 75 | [21] |
| 1 | 81 | 74 | [21] |
| 1 | 81 | 72 | [21] |

For this study, we use the $CO_2$ liquefaction process and electricity requirement of 89 kWh/ton $CO_2$ from [18] as it produces liquified $CO_2$ at a pressure of 20 bars. A pressure of 20 bars is below the maximum allowable working pressure (MAWP) of 24 bar for transporting liquid $CO_2$ by road using semi-trailer lorries,[22-24] which is the transportation mode used in this analysis. The reader can refer [18] for further details on the liquefaction plan.

**$CO_2$ Transportation**

We assume the liquified $CO_2$ is transported by road using semi-trailer trucks. The specifications for the semi-trailer truck are obtained from a commercial trailer manufacturer (Supplementary Table 7). For the onward trip to the cement plant where $CO_2$ is utilized, the transport of 1 kg of liquid $CO_2$ necessitates the transport of an additional 0.4 kg of tare weight. For the return trip, we account for the $CO_2$ emissions from transporting only the 0.4 kg of tare weight as the $CO_2$ is utilized.

**Supplementary Table 7 Specifications for road transportation of liquified $CO_2$.**

| | $CO_2$ payload (kg) | Tare weight (kg) | Total vehicle weight (kg) | Reference |
|---|---|---|---|---|
| ASCO $CO_2$ Semi-Trailer 25m$^3$ (882.9ft$^3$) PUR | 24,627 | 10,373 | 35,000 | [22] |

The $CO_2$ emissions from transporting $CO_2$ using a semi-trailer lorry is modeled based on the Ecoinvent dataset for a freight lorry with a capacity *greater* than 32 tonnes (as vehicle weight is 35 tons in Supplementary Table 7) and is equal to 112 g $CO_2$ per ton km, respectively (Supplementary Table 11).

The $CO_2$ is assumed to be transported for 810 km, which is the longest pipeline distance for $CO_2$ transport in the U.S.[2]

**$CO_2$ Vaporization**

The $CO_2$, which is in a liquified state, needs to be vaporized to a gaseous state and injected into the concrete sample for curing or mixing. The electricity required for $CO_2$ vaporization is obtained from a review of commercial $CO_2$ vaporizers (Supplementary Table 8). We use the lowest value of 5.3 kWh/ton $CO_2$ for $CO_2$ vaporization, which is reported in [25]

**Supplementary Table 8 Electricity required for $CO_2$ vaporization**

| Manufacturer | Vaporizer Capacity (kg/hour) | Electricity for liquid $CO_2$ vaporization (kWh/ton $CO_2$) | Reference |
|---|---|---|---|
| Asco | 1000 | 5.3 | [25] |
| Tomco | 1533 | 80 | [26] |
| Acme Cryogenics | 2040 | 88 | [27] |

**Supplementary Table 9 Electricity requirement and $CO_2$ emissions from $CO_2$ and steam curing of concrete**

| Electricity requirement and $CO_2$ emissions from $CO_2$ and stream curing of concrete | Value Used | Source |
|---|---|---|
| **Electricity requirement for $CO_2$ curing:** Power consumed for $CO_2$ curing of 1 $m^3$ of concrete | 38.8 kW/$m^3$ | [28,29] |
| **Steam curing:** $CO_2$ emissions from conventional steam curing | 39.55 kg $CO_2$/$m^3$/hour of curing<br><br>The study does not report the number of hours of steam curing over which the 39.55 kg of $CO_2$ is emitted. We use a conservative estimate and assume that 39.55 kg of $CO_2$ is emitted from 1 hour of steam curing. As a result, the above value is multiplied by the number of hours of steam curing to obtain the total value of $CO_2$ emissions from steam curing. | 9.91 kg of heavy oil + 10.35 kWh, which corresponds to 38.5 kg $CO_2$/$m^3$ (in Table 4[30])<br><br>An electricity $CO_2$ intensity value of 0.407 kg/kWh was used in [30], which we replace with the US average grid factor 0.499 kg/kWh[1] and obtain 39.55 kg $CO_2$/$m^3$ |

## 4. System Boundary Expansion to Determine the $CO_2$ emissions from Steel Slag and Fly Ash

**Steel Slag:** The production of 22.3 million tons of pig iron generated 2.9 million tons of granulated blast furnace slag as a by-product in the US in 2016.[31] Based on the above-mentioned ratio, 1 kg of slag is a by-product of producing 7.7 kg of pig iron. Therefore, the $CO_2$ impact per kg of slag is

$$\varphi_{SCM} = 7.7 * \varphi_{IO}$$

$\varphi_{IO}$ is the life cycle $CO_2$ emission from producing 1 kg of pig iron and is determined to be 2.2 kg $CO_2$/kg pig iron in SimaPro (using the ecoinvent dataset "Pig iron GLO| production | Alloc Def, U")

**Fly Ash:** In 2017, 664 $x10^9$ kg of coal was combusted[32] to generate $1.2x10^{12}$ kWh of electricity in the US[33], which gives a ratio of 0.55 kg coal combusted per kWh of electricity generated. In addition, $62.3x10^9$ kg of fly ash was produced[34] as a by-product of coal combustion and 702 $x10^9$ kg of coal was produced[35] in the US in 2017, which gives a ratio of 0.08 kg fly ash produced per kg coal combusted. Based on the above-mentioned ratios, 1 kg of fly ash is produced as a by-product when 22.7 kWh of coal electricity is generated. Therefore, the $CO_2$ impact per kg of fly ash is

$$\varphi_{SCM} = 22.7 * \varphi_{Elec\_Coal} * \alpha_{Cap}$$

$\varphi_{Elec\_Coal}$ is the life cycle $CO_2$ emission from producing 1 kWh of coal electricity is determined to be 1.2 kg $CO_2$/kWh in SimaPro (using the ecoinvent dataset "Electricity, high voltage WECC, US only| electricity production, hard coal | Alloc Def, U").

$\alpha_{Cap}$ is 0.1 if $CO_2$ is captured from a coal plant (because 90% of the emissions are captured) and used in CCU concrete production. $\alpha_{Cap}$ is 1 if there is no carbon capture from a coal plant in scenarios when (i) baseline concrete is produced with no carbon capture or (ii) $CO_2$ is captured from a combined cycle natural gas plant and used in CCU concrete production.

## 5. Economic Value-based Allocation to Determine the $CO_2$ Emissions from Steel Slag and Fly Ash

### Economic Value-based Allocation to Determine the $CO_2$ Emissions from Steel Slag

The price of steel slag is \$26/ton[36] and the price of pig iron price is \$410/ton[37]. 1 kg of slag is produced as a by-product when 7.7 kg of iron-ore is produced.

Therefore, the allocation factor based on economic value of the co-products pig iron and steel slag is 0.008 (i.e. [1x0.026]/[(1x0.026)+(7.7x0.41)])

Therefore, $CO_2$ intensity of steel slag using economic allocation is

$$\varphi_{SCM\_slag} = 0.008 * 7.7 * \varphi_{IO}$$

### Economic Value-based Allocation to Determine the $CO_2$ Emissions from Fly Ash

The price of fly ash price is \$50/ton[38] and the life cycle cost of electricity from coal is \$0.10/kWh[39]. 1 kg of fly ash is produced as a by-product when 22.7 kWh of coal electricity is generated.

Therefore, the allocation factor based on economic value of the co-products fly ash and coal electricity is 0.02 (i.e. (1x0.05)/((1x0.05)+(22.7x0.1)))

Therefore, $CO_2$ intensity of fly ash using economic allocation is

$$\varphi_{SCM\_ash} = 0.02 * 22.7 * \varphi_{Elec\_Coal} * \alpha_{Cap}$$

$\alpha_{Cap}$ is 0.1 if $CO_2$ is captured from a coal plant and used in CCU concrete production. $\alpha_{Cap}$ is 1 if there is no carbon capture from a coal plant i.e. when baseline concrete is produced with no carbon capture or when $CO_2$ is captured from a combined cycle natural gas plant and used in CCU concrete production.

## 6. Mass Based Allocation to Determine the $CO_2$ Emissions from Steel Slag and Fly Ash

**Mass Based Allocation to Determine the $CO_2$ Emissions from Steel Slag**

1 kg of slag is produced as a by-product when 7.7 kg of iron-ore is produced. Therefore, the allocation factor for slag based on mass of the co-products slag and iron ore is 1/(1+7.7) = 0.11

$CO_2$ intensity of steel slag using mass allocation is

$$\varphi_{SCM\_slag} = 0.11 * 7.7 * \varphi_{IO}$$

**Mass Based allocation to Determine the $CO_2$ Emissions from Fly Ash**

The mass allocation factor for fly ash based on mass of the co-products fly ash and coal production is 0.06.[40]

Therefore, $CO_2$ intensity of fly ash using mass allocation is

$$\varphi_{SCM\_ash} = 0.06 * 22.7 * \varphi_{Elec\_Coal} * \alpha_{Cap}$$

$\alpha_{Cap}$ is 0.1 if $CO_2$ is captured from a coal plant and used in CCU concrete production. $\alpha_{Cap}$ is 1 if there is no carbon capture from a coal plant i.e. when baseline concrete is produced with no carbon capture or when $CO_2$ is captured from a combined cycle natural gas plant and used in CCU concrete production.

## 7. Transportation Distance for Materials used to Produce CCU and Conventional Concrete

The transportation distance for materials used in the CCU and conventional concrete mix are assumed to be equal to the national average transportation distances in the US concrete industry.[41]

**Supplementary Table 10 Transportation Distance for Materials used to Produce CCU and Conventional Concrete**

| Transportation Mode | Portland Cement (km) | Fly Ash (km) | Slag Cement (km) | Crushed Coarse Aggregate (km) | Crushed Fine Aggregate (km) | Water (km) |
|---|---|---|---|---|---|---|
| Road | 124 | 107 | 47 | 31 | 7 | 124 |
| Rail | 78 | 98 | 10 | 32 | 1 | 78 |
| Ocean Ship | 116 | 22 | 104 | 12 | 0 | 116 |
| Barge | 54 | 0 | 37 | 3 | 0 | 54 |

The transportation distance for water is not reported in [41] and is assumed to be equal to the transportation distance for cement.

**Supplementary Table 11 Transportation $CO_2$ emissions by mode**

| Transportation Mode | $CO_2$ emissions - Mean (kg $CO_2$ per tonne-km) | EcoInvent Dataset used in SimaPro to determine $CO_2$ emissions |
|---|---|---|
| Road | 0.112 | Transport, freight, lorry > 32 metric ton, EURO5 (GLO)\| market for \| Alloc Def, U |
| Rail | 0.061 | Transport, freight train US\| market for \| Alloc Def, U |
| Ocean Ship | 0.006 | Transport, freight, sea, transoceanic tanker GLO\| market for \| Alloc Def, U |
| Barge | 0.048 | Transport, freight, inland waterways, barge tanker GLO\| market for \| Alloc Def, U |

## 8. Algorithm and Code Used to Determine the Results

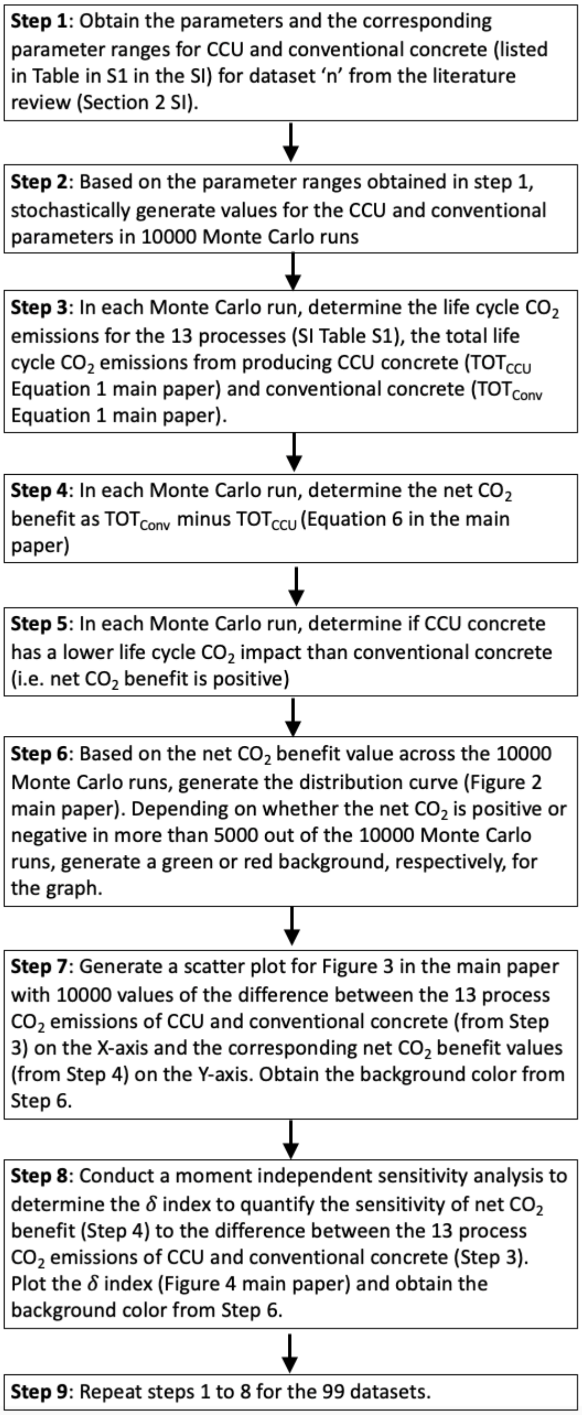


**Supplementary Figure 7 Algorithm to determine the net $CO_2$ benefit distribution, scatter plot and the δ index values.**

The code can be downloaded from [42]

## 9. High-Resolution Images for the Scatter Plot

The high-resolution images of the results can be downloaded from the links provided in Supplementary Table 12.

**Supplementary Table 12 High-resolution images of the results**

| Item No | Graph type | File name with Download Link |
|---|---|---|
| 1 | Scatter plot for the 13 individual processes | https://bit.ly/3hH3zqI |

**Visibility of the datapoints for the 13 processes in the scatter plots**: In the scatter plot, the limits of the x-axis are determined by the process with the greatest $CO_2$ emissions. For example, in Figure 4 in the main paper, the lower and the upper limit of the x-axis in plot 10 is -10 to +10, which is driven by the $CO_2$ emissions from OPC production (purple scattered points for process 1). At these limits of the x-axis, the scattered points corresponding to processes with significantly lower $CO_2$ emissions cannot be seen on the plot. For example, the maximum and minimum values for the x-axis for process 2 (coarse aggregate production) is -0.05 to +0.05 and, as a result, cannot be visualized on the x-axis ranging between -10 and +10. We have generated the scatter plot for each of the individual 13 processes in item 1 in Supplementary Table 12.

## 10. Scenario Analysis – CCU Concrete Production when $CO_2$ is Captured from a Natural Gas Combined Cycle (NGCC) Plant

The net $CO_2$ benefit of CCU concrete production with $CO_2$ captured from a NGCC plant is negative with a likelihood of 50% or greater in 61, 65 or 68 out of the 99 datasets when system boundary expansion (Supplementary Figure 8), mass-based allocation (Supplementary Figure 9) or economic allocation (Supplementary Figure 10) is used, respectively.

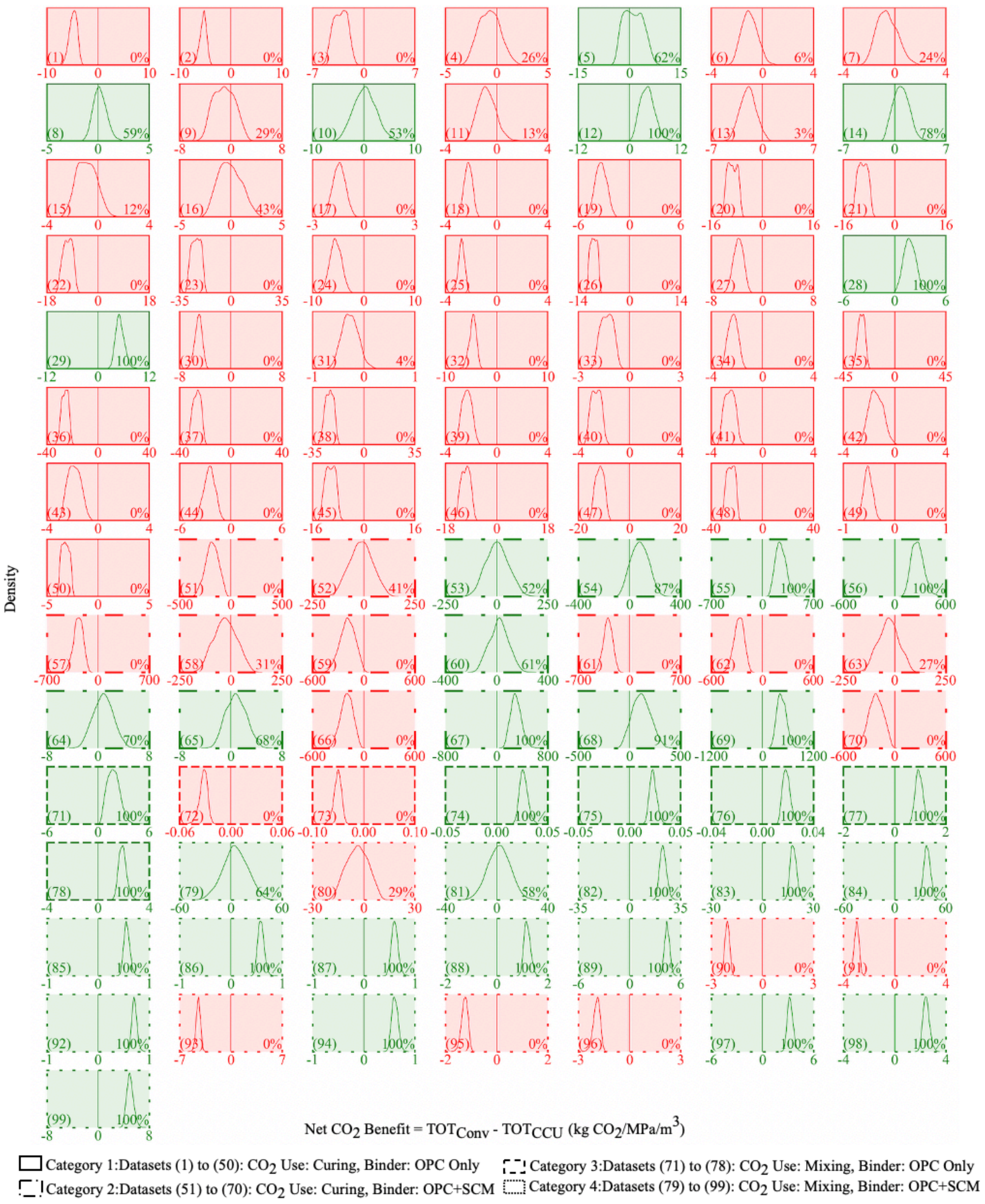


**Supplementary Figure 8 The net $CO_2$ benefit of CCU concrete production across 99 datasets when $CO_2$ is captured from a NGCC power plant and system boundary expansion is used to account for the $CO_2$ emissions from producing the supplementary cementitious**

**materials. The net $CO_2$ benefit of CCU concrete is negative with likelihood of 50% or more in 61 of the 99 datasets (red plots).**

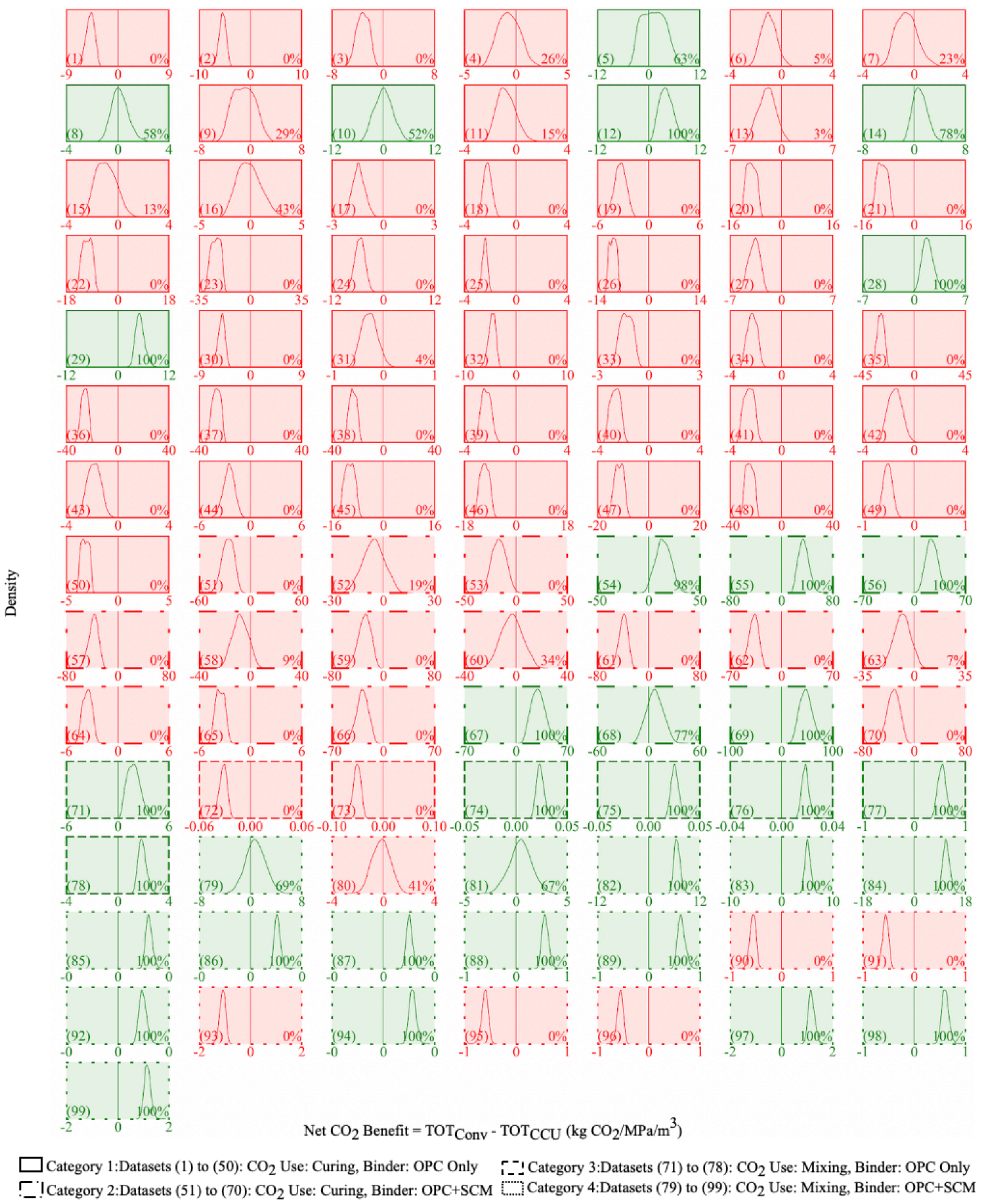

**Supplementary Figure 9 The net $CO_2$ benefit of CCU concrete production across 99 datasets when $CO_2$ is captured from a NGCC power plant and mass-based allocation is used to account for the $CO_2$ emissions from producing the supplementary cementitious materials. The net $CO_2$ benefit of CCU concrete is negative with likelihood of 50% or more in 65 of the 99 datasets (red plots).**

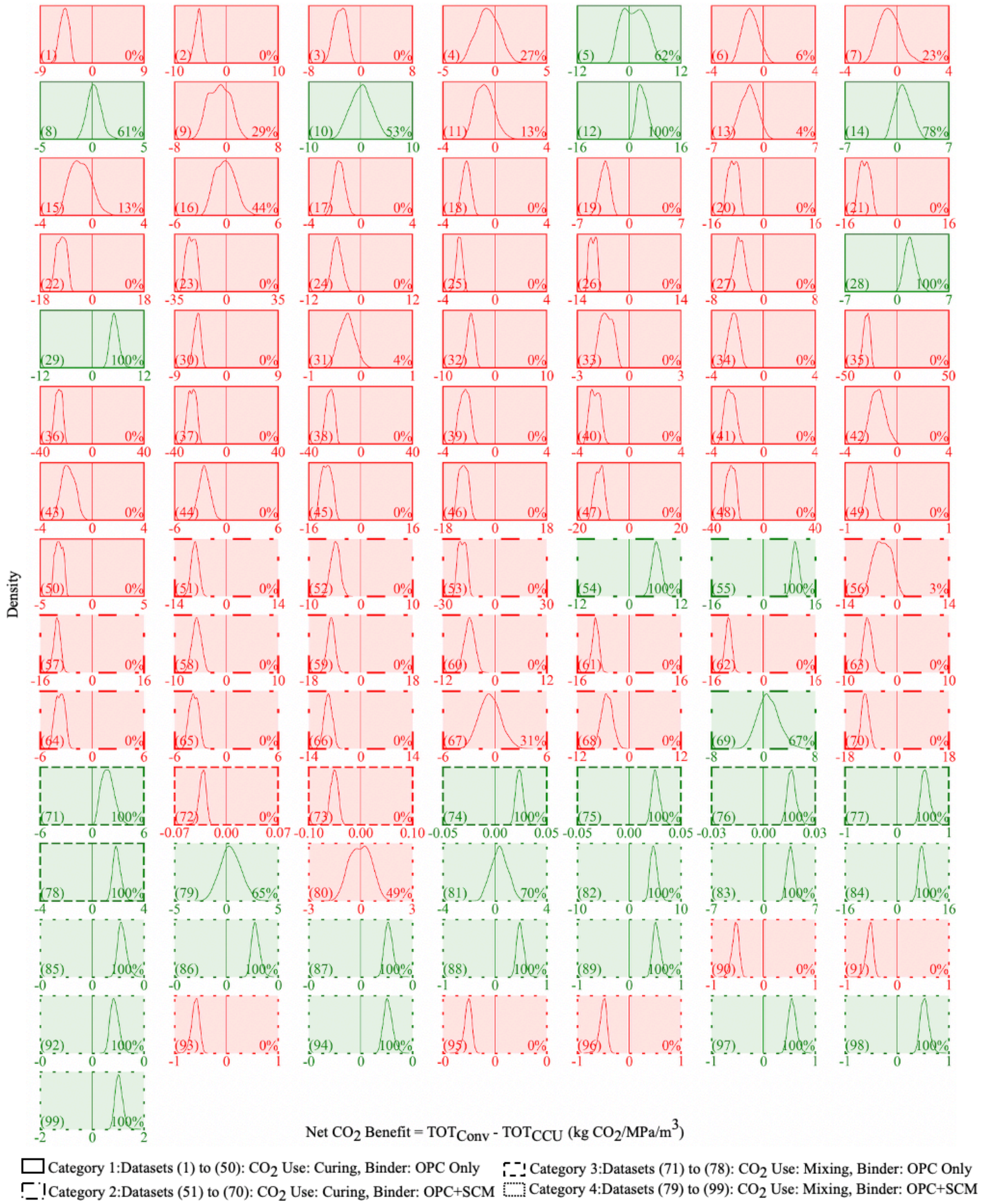


**Supplementary Figure 10 The net $CO_2$ benefit of CCU concrete production across 99 datasets when $CO_2$ is captured from a NGCC power plant and economic allocation is used**

**to account for the $CO_2$ emissions from producing the supplementary cementitious materials. The net $CO_2$ benefit of CCU concrete is negative with likelihood of 50% or more in 68 of the 99 datasets (red plots).**

## 11. Scenario Analysis – CCU Concrete Production using $CO_2$ Captured from a Cement Plant

In this scenario, the $CO_2$ is captured from a cement plant and used in the curing of concrete (hereafter "$CO_2$ captured rom cement plant" scenario). The system boundary diagram normalizes processes, which are specific to CCU concrete production (in green in Supplementary Figure 11 in SI), to the mass of $CO_2$ captured and used in the mixing or curing of concrete.

There are three differences between the "$CO_2$ captured from cement plant" and the baseline scenario (main paper Figure 1).

1. In the CCU pathway, the $CO_2$ captured from the cement plant is in excess (by X kg in Supplementary Figure 11) of the $CO_2$ required for curing/mixing in concrete (Y kg in Supplementary Figure 11). The X kg of captured $CO_2$ is calculated from the mass of cement produced and used in concrete production (obtained from the dataset). As a result, the system producing the CCU concrete has to account for the X kg of excess captured $CO_2$. The excess X kg of $CO_2$ can be utilized in the production of alternate CCU products (e.g. chemicals, fuels) or can be sequestered. A complete exploration of the range of possibilities to utilize the excess X kg of captured $CO_2$ is beyond the scope of this research.

   To account for this excess captured $CO_2$ feedstock in the CCU pathway, we include a coal power plant in the conventional pathway which generates the electricity ($E_x$ kWh)

required to capture the X kg of $CO_2$. As a result, the conventional pathway now produces 1 MPa of concrete, X kg of captured $CO_2$ and $E_x$ kWh of electricity.

To account for the excess electricity of $E_x$ kWh, a coal plant is operated without carbon capture to generate an electricity of $E_x$ kWh in the CCU pathway.

2. We assume the $CO_2$ transportation distance is zero as $CO_2$ is captured and utilized in the cement plant. Therefore, we exclude $CO_2$ transportation and consider a total of 12 processes instead of the 13 processes in the baseline scenario (Figure 1 main paper).
3. The energy penalty for capturing $CO_2$ from a cement plant is obtained from an International Energy Agency (IEA) study which specifically analyzes $CO_2$ capture from a cement plant.[43] The IEA study reported that 26,255 MWh/y of electricity (Table 4-2 in [43]) is used in a post-combustion capture unit to capture 1,067,734 t/y of $CO_2$ (Table 4-3 in [43]) from a cement plant. As a result, the electricity required to capture $CO_2$ is 20 kWh/ton captured $CO_2$. To this value, we add the electricity requirement of 89 kWh/ton $CO_2$ for $CO_2$ liquefaction (Section 3 "$CO_2$ Liquefaction") to obtain a total electricity penalty of 109 kWh/ton $CO_2$ to capture and compress $CO_2$ from a cement plant.
4. The $CO_2$ intensity of the Ordinary Portland Cement (OPC) used in CCU concrete is 90% lower than conventional concrete as 90% of the $CO_2$ is captured from the cement plant in the CCU concrete system.

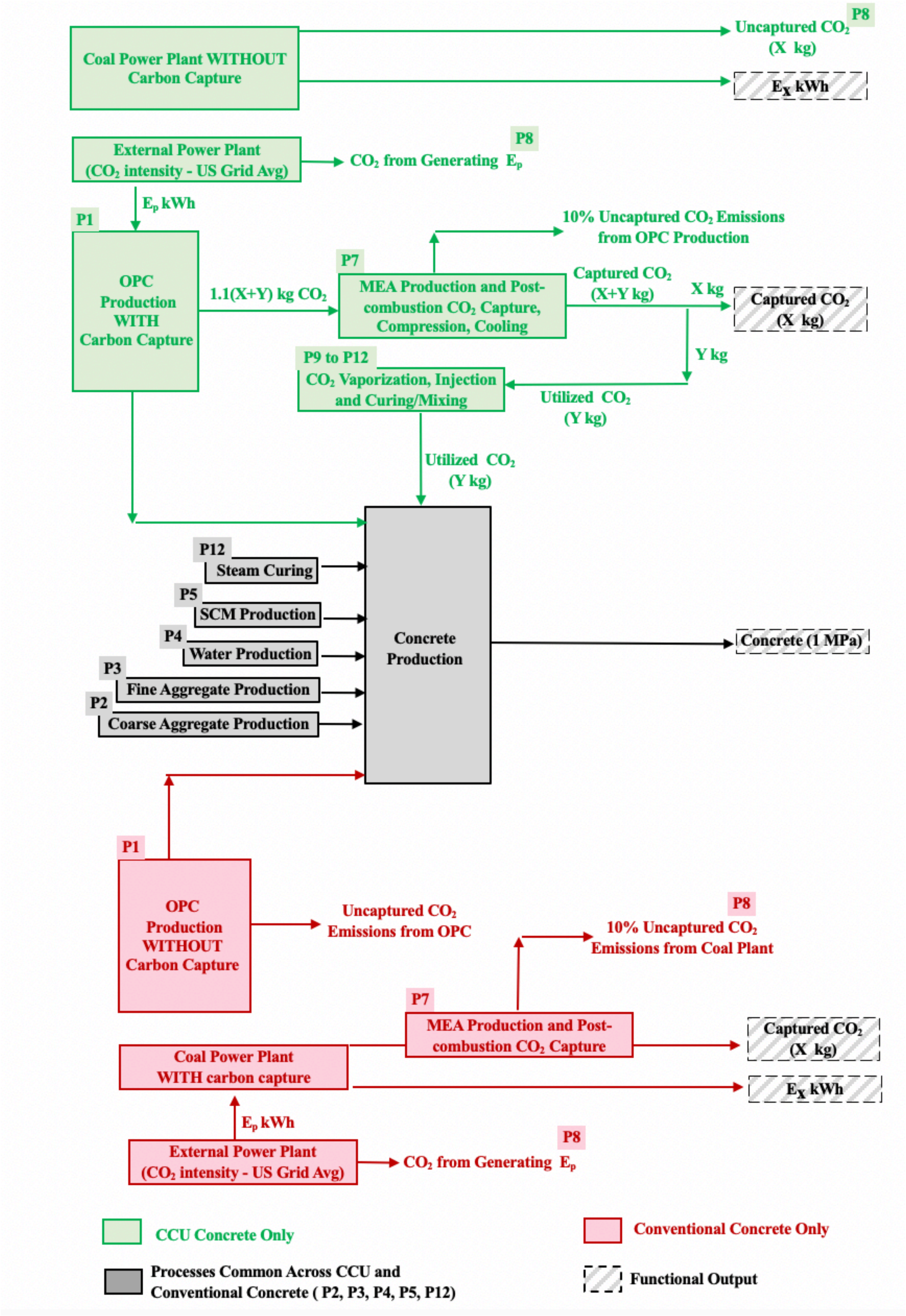

Coal Power Plant WITHOUT Carbon Capture
Uncaptured $CO_2$ (X kg)
P8
$E_X$ kWh
External Power Plant ($CO_2$ intensity - US Grid Avg)
$CO_2$ from Generating $E_p$
P8
$E_p$ kWh
P1
OPC Production WITH Carbon Capture
1.1(X+Y) kg $CO_2$
P7
MEA Production and Post-combustion $CO_2$ Capture, Compression, Cooling
10% Uncaptured $CO_2$ Emissions from OPC Production
Captured $CO_2$ (X+Y kg)
X kg
Captured $CO_2$ (X kg)
Y kg
P9 to P12
$CO_2$ Vaporization, Injection and Curing/Mixing
Utilized $CO_2$ (Y kg)
Utilized $CO_2$ (Y kg)
P12
Steam Curing
P5
SCM Production
P4
Water Production
P3
Fine Aggregate Production
P2
Coarse Aggregate Production
Concrete Production
Concrete (1 MPa)
P1
OPC Production WITHOUT Carbon Capture
Uncaptured $CO_2$ Emissions from OPC
P8
10% Uncaptured $CO_2$ Emissions from Coal Plant
P7
MEA Production and Post-combustion $CO_2$ Capture
Coal Power Plant WITH carbon capture
Captured $CO_2$ (X kg)
$E_X$ kWh
$E_p$ kWh
External Power Plant ($CO_2$ intensity - US Grid Avg)
$CO_2$ from Generating $E_p$
P8
CCU Concrete Only
Conventional Concrete Only
Processes Common Across CCU and Conventional Concrete ( P2, P3, P4, P5, P12)
Functional Output

**Supplementary Figure 11 System boundary diagram to quantify the net $CO_2$ benefit of CCU concrete production when $CO_2$ is captured from a cement plant and used in concrete production.**

When $CO_2$ is captured from a cement plant and used in concrete production, CCU concrete has lower life cycle $CO_2$ emissions than conventional concrete with at least a 50% likelihood in 55 out of the 99 datasets (plots with green background in Supplementary Figure 12). In the baseline scenario, CCU concrete has lower life cycle $CO_2$ emissions than conventional concrete with at least a 50% likelihood in 43 out of the 99 datasets.

The difference in the results can be attributed to the decreasing influence of OPC production (due to $CO_2$ capture in the cement plant) on the net $CO_2$ benefit. Datasets which were previously red in the baseline scenario due to lower OPC requirements in CCU concrete than in conventional concrete (e.g. 9, 11 and 15 in Figure 5 main paper) are now green due to decreased contribution of $CO_2$ from OPC production to the net $CO_2$ benefit.

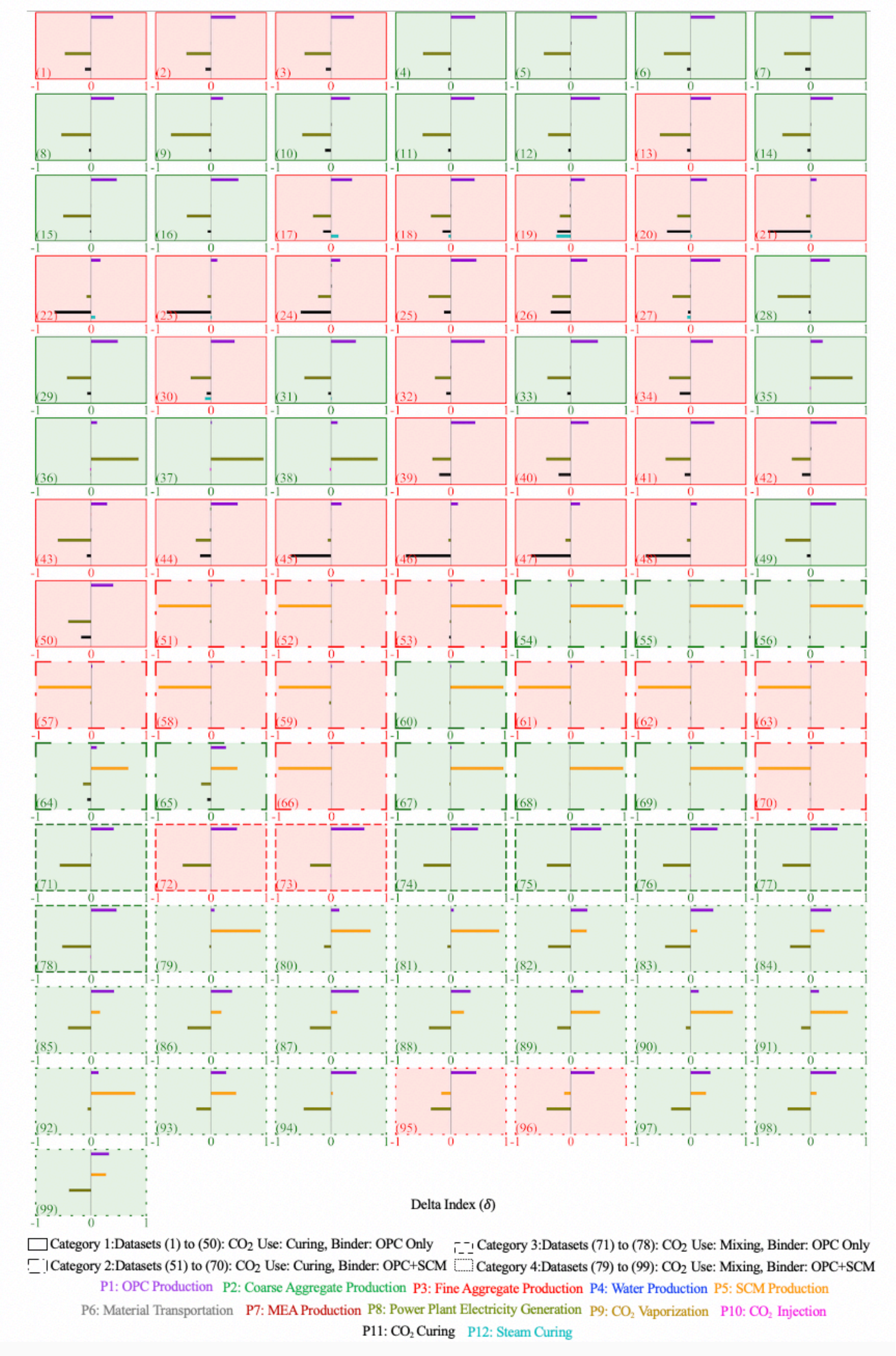
Delta Index (δ)
Category 1:Datasets (1) to (50): $CO_2$ Use: Curing, Binder: OPC Only
Category 2:Datasets (51) to (70): $CO_2$ Use: Curing, Binder: OPC+SCM
Category 3:Datasets (71) to (78): $CO_2$ Use: Mixing, Binder: OPC Only
Category 4:Datasets (79) to (99): $CO_2$ Use: Mixing, Binder: OPC+SCM
P1: OPC Production
P2: Coarse Aggregate Production
P3: Fine Aggregate Production
P4: Water Production
P5: SCM Production
P6: Material Transportation
P7: MEA Production
P8: Power Plant Electricity Generation
P9: $CO_2$ Vaporization
P10: $CO_2$ Injection
P11: $CO_2$ Curing
P12: Steam Curing

**Supplementary Figure 12 δ indices quantifying influence of the difference between the $CO_2$ emissions of the 12 contributing processes of CCU and conventional concrete on the net $CO_2$ benefit when $CO_2$ is captured from a cement plant and utilized for concrete production.**

## 12. Scenario Analysis – CCU Concrete Production when $CO_2$ Emissions from $CO_2$ Transport is Zero

To simulate zero $CO_2$ impact from $CO_2$ transport, we assume the transportation is zero km instead of the 810 km in the baseline scenario ($D_T$=0 km in Process 9 in Supplementary Table ***1***). The results (Supplementary Figure 13 in the SI) show that CCU concrete has a lower life cycle $CO_2$ emission than conventional concrete in 44 of the 99 datasets (green plots), which is similar to 43 of the 99 datasets in the baseline scenario (Figure 3 in the main paper).

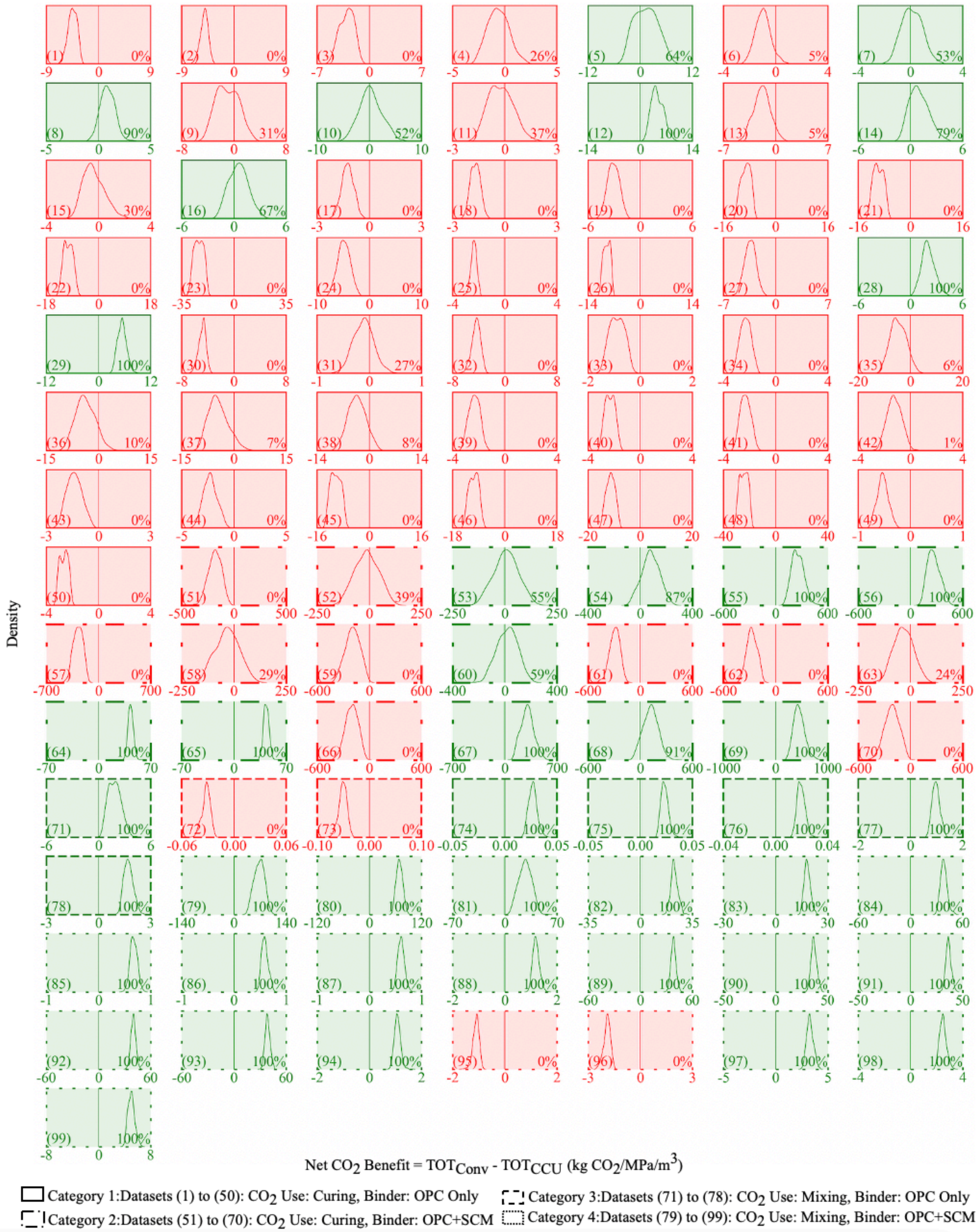


**Supplementary Figure 13 The net $CO_2$ benefit of CCU concrete production across 99 datasets when the distance over which the captured $CO_2$ is transported is zero. The curve**

**in each plot represents the distribution of the net $CO_2$ benefit, which is the difference between the total $CO_2$ emissions from producing conventional and CCU, across 10000 Monte Carlo runs. When the net $CO_2$ benefit is negative in at least 5000 of the 10000 Monte Carlo runs (50% likelihood), the background is red. If the background is green, then it signifies that the net $CO_2$ benefit is positive with a likelihood greater than 50%.**

## 13. Literature Review to Identify Knowledge Gaps and Shortcomings

The literature review covered 23 studies containing claims on the $CO_2$ benefit of CCU concrete. The studies with reference numbers can be found here: https://rb.gy/afq1te

The Venn diagram represents the organization of literature review to identify knowledge gaps and shortcomings when determining the $CO_2$ impact of CCU concrete.

The literature review is organized based on whether a study accounts for the following 5 aspects when quantifying the net $CO_2$ impact of CCU concrete

- **$CO_2$ impact of capturing, transporting, compressing and utilizing $CO_2$:** The $CO_2$ emissions from the upstream processes of capturing, transporting, compressing and utilizing $CO_2$ will decrease the net $CO_2$ benefit from CCU concrete. Only 3 (A[1] + E[2]) out of the 23 studies account for the upstream processes. However, none of the 3 studies account for data uncertainty and only 1 study accounts for change in compressive strength. None of the 3 studies account for the compensation of the energy penalty of $CO_2$ capture and allocation of $CO_2$ emissions to the supplementary cementitious materials (SCM) (SI Section 6), which are by-products of coal electricity and pig-iron production

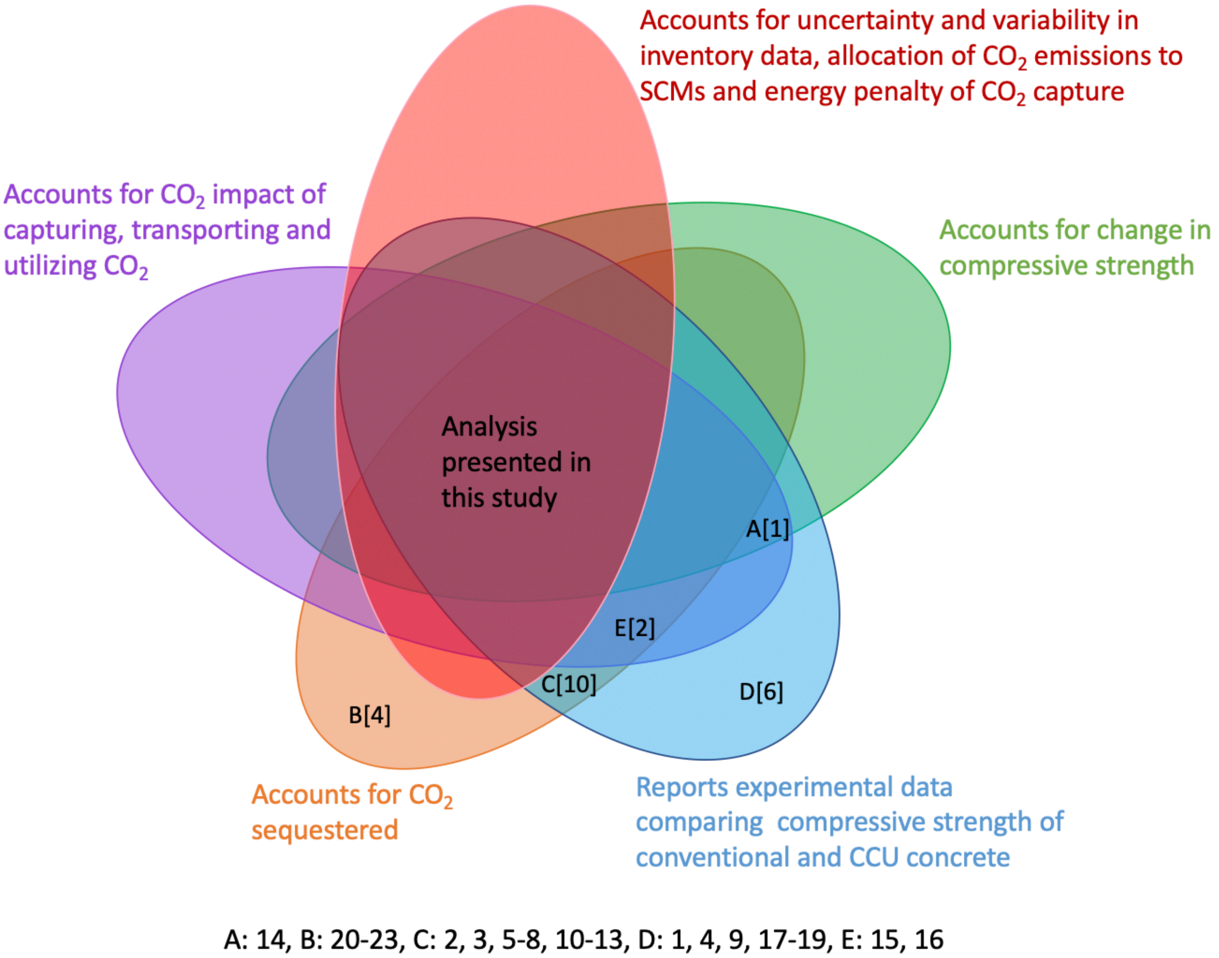


**Supplementary Figure 14 The 5 ovals in the Venn diagram represent 5 key aspects in a study that are required to determine and robustly substantiate the claims on the $CO_2$ impact of CCU concrete on a life cycle basis. The numbers within the square parenthesis represent the number of studies in 5 regions of the Venn diagram (A, B, C, D and E). The studies corresponding to the parenthesized numbers for the 5 regions are listed below the Venn diagram. For example, studies 15 and 16 are the 2 studies in region E (i.e. E[2]) of the Venn Diagram.**

- **uncertainty and variability in data, allocation for supplementary cementitious materials (SCM) and compensating for energy penalty:** As observed in SI Section 2,

there is significant variability in the materials used in design mixes across the studies. In addition, there is uncertainty in the life cycle data which will introduce uncertainty in the $CO_2$ footprint of the material and energy inventory. For example, the uncertainty in the life cycle $CO_2$ footprint of the material components used in the concrete is presented in Supplementary Table 1.

The SCMs - fly ash and slag - are by-products of coal electricity generation and pig-iron production. As a result, when determining the $CO_2$ footprint of SCMs used in CCU concrete, the analysis should account for the allocation of $CO_2$ emissions between (i) flash ash and coal electricity and (ii) slag and pig-iron (SI Table S1 Process 5).

The capture of $CO_2$ (to be utilized in CCU concrete production) from a power plant incurs an energy penalty (SI Table S1 Process 8). The $CO_2$ emitted from generating electricity from an external power plant to compensate for energy penalty needs to be accounted for when determining the $CO_2$ impact of CCU concrete.

None of the studies account for data uncertainty, allocation of $CO_2$ emissions between the by-products SCM and coal electricity and steel, and the energy penalty of $CO_2$ capture.

- **change in compressive strength when quantifying the climate benefit of CCU concrete:** Ordinary Portland Cement (OPC) is the most $CO_2$ intensive material component in concrete. The increased use of OPC to compensate for loss in compressive strength in CCU concrete can significantly increase the $CO_2$ footprint of CCU concrete on a life cycle basis.

  As shown in Supplementary Figure 14, only 1 (A[1]) of the 23 studies accounts for the change in compressive strength. However, this study does not account for data

uncertainty, allocation of $CO_2$ emissions between the by-products SCM and coal electricity and steel, and the energy penalty of $CO_2$ capture.

- **experimental data comparing compressive strength of conventional and CCU concrete:** This helps distinguish between studies that quantify the $CO_2$ impact of CCU concrete from (i) inventory data collected from experiments comparing the compressive strengths of CCU and conventional concrete and (ii) a review of other studies. The net $CO_2$ benefit of CCU concrete determined from primary experimental data is more robust as (i) there is clarity and transparency in system boundary assumptions and inventory items consumed (ii) $CO_2$ hotspots are identified based on inventory requirements and (iii) strategies to address the hotpot can be directed towards reducing the consumption of inventory items contributing the most to the $CO_2$ footprint of CCU concrete. 19 out the 23 studies (A[1] + C[10] + D[6] + E[2]) report primary experimental data. However, among the 19 studies, there is no study that accounts for data uncertainty, only 1 study accounts for change in compressive strength (A[1]) and 3 studies (A[1]+E[2]) account for the $CO_2$ impact of capture, transport and utilization of $CO_2$.
- **$CO_2$ sequestered through concrete curing or mixing:** If a study quantifies the climate benefit based on only the $CO_2$ sequestered, it is not accounting for the $CO_2$ impact from the change in compressive strength and the upstream processes related to capture, transport and utilization of $CO_2$. The inclusion of the $CO_2$ impact of the upstream processes decreases the net $CO_2$ benefit of CCU concrete.

  As seen in Supplementary Figure 14, 17 studies account for $CO_2$ sequestered (A[1] + B[4] + C[10] + E[2]). Only 2 (E[2]) of the 17 studies account for the $CO_2$ impact of capturing, transporting and utilizing $CO_2$. None of the 17 studies account for data

uncertainty, change in compressive strength of CCU concrete, compensation of the energy penalty of $CO_2$ capture or allocation of $CO_2$ emissions to the SCMs.

Due to the above knowledge gaps and methodological shortcomings, the findings from one study cannot be generalized to other CCU studies and there is no comprehensive assessment to generalize and support claims on the net $CO_2$ impact of CCU concrete. We further illustrate the shortcomings though three examples from the reviewed literature

1. **Example 1 - Study number 14 in region A of the Venn diagram (Monkman, S., & MacDonald, M., 2017)[3]**: The study uses point values for material and energy inventory items and does not account for the variability in inventory data and uncertainty in life cycle data when quantifying the net $CO_2$ benefit of CCU concrete. Moreover, this study investigates $CO_2$ mixing in concrete and, therefore, the findings are not applicable to $CO_2$ curing of concrete. In addition, the study does not account for the (i) allocation of the $CO_2$ emissions between the supplementary cementitious materials (SCMs) which are by-products of coal electricity and steel production and the (ii) compensation of the energy penalty associated with carbon capture.
2. **Examples 2 and 3 - Study number 21 (Sanna et al)[44] and study number 20 (Hepburn et al)[45] in region B of the Venn diagram**: The 2 studies quantify the $CO_2$ benefit based on the $CO_2$ sequestered in concrete and do not account for the other 4 aspects in Supplementary Figure 14. As a result, the 2 studies do not quantify the net $CO_2$ benefit of CCU concrete production on a life cycle basis.

## Supplementary References


1 Skone, T. J. *et al.* Carbon Dioxide Utilization Life Cycle Analysis Guidance for the U.S. Carbon Use and Reuse Program. *National Energy Technology Laboratory,* (2019).

2 U.S. Department of Energy. A Review of the CO2 Pipeline Infrastructure in the U.S. (2015).

3 Monkman, S. & MacDonald, M. On carbon dioxide utilization as a means to improve the sustainability of ready-mixed concrete. *Journal of Cleaner Production* **167**, 365-375, doi:10.1016/j.jclepro.2017.08.194 (2017).

4 Monkman, S., MacDonald, M., Hooton, R. D. & Sandberg, P. Properties and durability of concrete produced using CO2 as an accelerating admixture. *Cement and Concrete Composites* **74**, 218-224, doi:10.1016/j.cemconcomp.2016.10.007 (2016).

5 Monkman, S. & MacDonald, M. Carbon dioxide upcycling into industrially produced concrete blocks. *Construction and Building Materials* **124**, 127-132, doi:10.1016/j.conbuildmat.2016.07.046 (2016).

6 Bare, J. TRACI 2.0: the tool for the reduction and assessment of chemical and other environmental impacts 2.0. *Clean Technologies and Environmental Policy* **13**, 687-696, doi:10.1007/s10098-010-0338-9 (2011).

7 Koornneef, J., van Keulen, T., Faaij, A. & Turkenburg, W. Life cycle assessment of a pulverized coal power plant with post-combustion capture, transport and storage of CO2. *International Journal of Greenhouse Gas Control* **2**, 448-467, doi:10.1016/j.ijggc.2008.06.008 (2008).

8 Carnegie Mellon University. Amine-based Post-Combustion CO2 Capture. (2019).

9 Intergovernmental Panel on Climate Change (IPCC). IPCC Special Report on Carbon dioxide capture and storage. (2005).

10 Boot-Handford, M. E. *et al.* Carbon capture and storage update. *Energy Environ. Sci.* **7**, 130-189, doi:10.1039/c3ee42350f (2014).

11 Rochelle, G. *et al.* Aqueous piperazine as the new standard for CO2 capture technology. *Chemical Engineering Journal* **171**, 725-733, doi:10.1016/j.cej.2011.02.011 (2011).

12 Reiter, G. & Lindorfer, J. Evaluating CO2 sources for power-to-gas applications – A case study for Austria. *Journal of CO2 Utilization* **10**, 40-49, doi:10.1016/j.jcou.2015.03.003 (2015).

13 Metz, B., Davidson, O., De Coninck, H. *Carbon dioxide capture and storage: special report of the intergovernmental panel on climate change*. (Cambridge University Press, 2005).

14 Peeters, A. N. M., Faaij, A. P. C. & Turkenburg, W. C. Techno-economic analysis of natural gas combined cycles with post-combustion CO2 absorption, including a detailed evaluation of the development potential. *International Journal of Greenhouse Gas Control* **1**, 396-417, doi:10.1016/s1750-5836(07)00068-0 (2007).

15 Korre, A., Nie, Z. & Durucan, S. Life cycle modelling of fossil fuel power generation with post-combustion CO2 capture. *International Journal of Greenhouse Gas Control* **4**, 289-300, doi:10.1016/j.ijggc.2009.08.005 (2010).

16 Rao, A., B. , Rubin, E., S. & Berkenpas, M., B. An integrated modelling framework for carbon management technologies. Technical Documentation: Amine-based CO2 Capture and Storage Systems for Fossil Fuel Power Plant. *Contract No. DE-FC26–00NT40935. Carnegie Melon University, USA,* (2004).

17 Aspelund, A., Mølnvik, M. J. & De Koeijer, G. Ship Transport of CO2: Technical solutions and analysis of costs, energy utilization, exergy efficiency and CO2 emissions. *Chemical Engineering Research and Design* **84**, 847-855, doi:10.1205/cherd.5147 (2006).

18 Deng, H., Roussanaly, S. & Skaugen, G. Techno-economic analyses of CO2 liquefaction: Impact of product pressure and impurities. *International Journal of Refrigeration* **103**, 301-315, doi:10.1016/j.ijrefrig.2019.04.011 (2019).

19 Seo, Y., Huh, C., Lee, S. & Chang, D. Comparison of CO 2 liquefaction pressures for ship-based carbon capture and storage (CCS) chain. *International Journal of Greenhouse Gas Control* **52**, 1-12, doi:10.1016/j.ijggc.2016.06.011 (2016).

20 Roussanaly, S., Skaugen, G., Aasen, A., Jakobsen, J. & Vesely, L. Techno-economic evaluation of CO 2 transport from a lignite-fired IGCC plant in the Czech Republic. *International Journal of Greenhouse Gas Control* **65**, 235-250, doi:10.1016/j.ijggc.2017.08.022 (2017).

21 Duan, L., Chen, X. & Yang, Y. Study on a novel process for CO2compression and liquefaction integrated with the refrigeration process. *International Journal of Energy Research* **37**, 1453-1464, doi:10.1002/er.2951 (2013).

22 ASCO. *Transportable CO2 Tank*, <https://www.ascoco2.com/fileadmin/PDF_Download/PDF_Produkte/PDF_CO2_und_Trockeneis_Zubehoer/CO2_Lagerung/en/ASCO_Transportable_CO2_Tanks.pdf> (2020).

23 TOMCO Systems. *Liquid CO2 21.2 ISO Container*, <https://tomcosystems.com/wp-content/uploads/2018/11/2.2.1.Liquid-CO2-ISO-Container.pdf> (2020).

24 Taylor-Wharton. *Vacuum-Jacketed CO2/N2O Tanks*, <https://twcryo.com/wp-content/uploads/2018/08/SCE-Series-TWM-B009-Rev00.pdf> (2020).

25 ASCO. *Atmospheric ASCO CO2 Vaporiser*, <https://www.ascoco2.com/fileadmin/PDF_Download/PDF_Produkte/PDF_CO2_und_Trockeneis_Zubehoer/Ergaenzendes_CO2_Zubehoer/en/ASCO_Atmospheric_CO2_Vaporisers.pdf> (2020).

26 TOMCO Systems. *Electric Direct to Process Vaporizer for Liquid Carbon Dioxide*, <https://tomcosystems.com/wp-content/uploads/2018/11/3.2.1.Applications-Equipment_DTP.pdf> (2020).

27 Acme Cryogenics. *Electric Vaporizers*, <https://www.acmecryo.com/product/electric-vaporizers/> (2020).

28 El-Hassan, H., Shao, Y., Ghouleh, Z. . Effect of initial curing on carbonation of lightweight concrete masonry units. *ACI Materials Journal* **110** (2013).

29 El-Hassan, H. & Shao, Y. Carbon Storage through Concrete Block Carbonation. *Journal of Clean Energy Technologies*, 287-291, doi:10.7763/jocet.2014.V2.141 (2014).

30 Kawai, K., Sugiyama, T., Kobayashi, K., Sano, S. Inventory Data and Case Studies for Environmental Performance Evaluation of Concrete Structure Construction. *Journal of Advanced Concrete Technology* **3**, 435-456 (2005).

31 U.S. Geological Survey (USGS). *Iron and Steel Slag Statistics and Information*, <https://www.usgs.gov/centers/nmic/iron-and-steel-slag-statistics-and-information?qt-science_support_page_related_con=0#qt-science_support_page_related_con> (2019).

32 U.S. Energy Information Administration (EIA). *Coal: Consumption for Electricity Generation*, <https://www.eia.gov/electricity/annual/> (2019).

33 U.S. Energy Information Administration (EIA). Total Electric Power Industry Summary Statistics, 2018 and 2017. (2019).

34 American Coal Ash Association. *Coal Ash Recycling Reaches Record 64 Percent Amid Shifting Production and Use Patterns*, <https://www.acaa-usa.org/Portals/9/Files/PDFs/Coal-Ash-Production-and-Use-2017.pdf> (2017).

35 U.S. Energy Information Administration (EIA). *Annual Coal Report*, <https://www.eia.gov/coal/annual/> (2017).

36 United States Geological Society. *Iron and Steel Slag*, <https://prd-wret.s3-us-west-2.amazonaws.com/assets/palladium/production/atoms/files/mcs-2019-fesla.pdf> (2018).

37 Bulletin, M. *Pig iron wrap: CIS exporters get higher prices*, <https://www.amm.com/Article/3811872/Pig-iron-wrap-CIS-exporters-get-higher-prices.html> (2020).

38 Boral Resources. *Fly Ash*, <http://acquia-prod.boral.com/sites/corporate/files/media/field_document/180528-Flyash-slides-for-investors-as-at-29May2018.pdf> (2018).

39 Lazard. *Levelized Cost of Energy and Levelized Cost of Storage*, <https://www.lazard.com/perspective/levelized-cost-of-energy-and-levelized-cost-of-storage-2018/> (2018).

40 Miller, S. A. Supplementary cementitious materials to mitigate greenhouse gas emissions from concrete: can there be too much of a good thing? *Journal of Cleaner Production* **178**, 587-598, doi:10.1016/j.jclepro.2018.01.008 (2018).

41 Athena Sustainable Materials Institute. NRMCA Member National and Regional Life Cycle Assessment Benchmark (Industry Average) Report. (2014).

42 Ravikumar, D., Zhang, D., Keoleian, G., Miller, S., Sick, V., Li, V.,. Carbon dioxide utilization in concrete curing or mixing might not produce a net climate benefit. *CCU_Concrete_v1.0*, doi:https://doi.org/10.5281/zenodo.4308983 (2020).

43 International Energy Agency. *CO2 Capture in the Cement Industry*, <https://ieaghg.org/docs/General_Docs/Reports/2008-3.pdf> (2008).

44 Sanna, A., Uibu, M., Caramanna, G., Kuusik, R. & Maroto-Valer, M. M. A review of mineral carbonation technologies to sequester CO2. *Chem Soc Rev* **43**, 8049-8080, doi:10.1039/c4cs00035h (2014).

45 Hepburn, C. *et al.* The technological and economic prospects for CO2 utilization and removal. *Nature* **575**, 87-97, doi:10.1038/s41586-019-1681-6 (2019).
